# Dispersive liquid-liquid microextraction coupled with ultra performance liquid chromatography-fluorescence detection for the determination of polycyclic aromatic hydrocarbons in tea: Method development, validation, greenness assessment, and dietary exposure risk assessment

**DOI:** 10.1016/j.fochx.2026.104387

**Published:** 2026-08-29

**Authors:** Kim Liu Tan, Jalal T. Althakafy, Yin-Hui Leong, Yong Shen Chua, Faiz Bukhari Mohd Suah, Yong Foo Wong

**Affiliations:** a Center for Research on Multidimensional Separation Science, School of Chemical Sciences, Universiti Sains Malaysia, 11800 Penang, Malaysia; b Department of Chemistry, Faculty of Science, Umm Al-Qura University, Makkah 21955, Saudi Arabia; c National Poison Centre, Universiti Sains Malaysia, 11800 Penang, Malaysia; d School of Chemical Sciences, Universiti Sains Malaysia, 11800 Penang, Malaysia

**Keywords:** Benz(*a*)anthracene, Benzo(*a*)pyrene, Benzo(*b*)fluoranthene, Chrysene, Fermented tea, Post-fermented tea, UPLC-FLD

## Abstract

This study presents an improved dispersive liquid-liquid microextraction with ultra performance liquid chromatography–fluorescence detection (DLLME-UPLC-FLD) method for determination of eight European Food Safety Authority-recommended polycyclic aromatic hydrocarbons (PAHs) in teas marketed in Malaysia. DLLME used dichloromethane as extraction solvent and methanol as dispersive solvent. Separation was effected using C18 column with FLD detection at 270 nm excitation and four emission wavelengths. The method demonstrated good linearity, detection limits (3.87–32.5 ng L^−1^), precision (≤9.91% RSD), recoveries (85.13–101.71%), enrichment factors (35–48), and greenness scores of 0.53 (AGREE). The analysis of 43 commercial tea infusions and tisanes showed chrysene was the most prevalent PAHs. The mean total concentration of ∑PAH8 followed the trend: post-fermented tea >non-fermented tea (31.61 ng kg^−1^) > fully fermented tea (17.10 ng kg^−1^) > semi-fermented tea (8.35 ng kg^−1^) > tisanes (4.28 ng kg^−1^). Margin of exposure values were above the critical safety threshold (10,000) for all categories.

## Highlights

• PAH8 in tea samples were analyzed using validated DLLME-UPLC-FLD.
• Chrysene was detected in all tea categories with a detection frequency of 100%.
• Post-fermented tea showed the highest mean concentration of ∑PAH8.
• Margin of exposure values indicated low dietary exposure to PAHs through tea consumption.
• The DLLME–UPLC–FLD method is simple, rapid, and environmentally friendly.

## Graphical abstract

**Figure:**
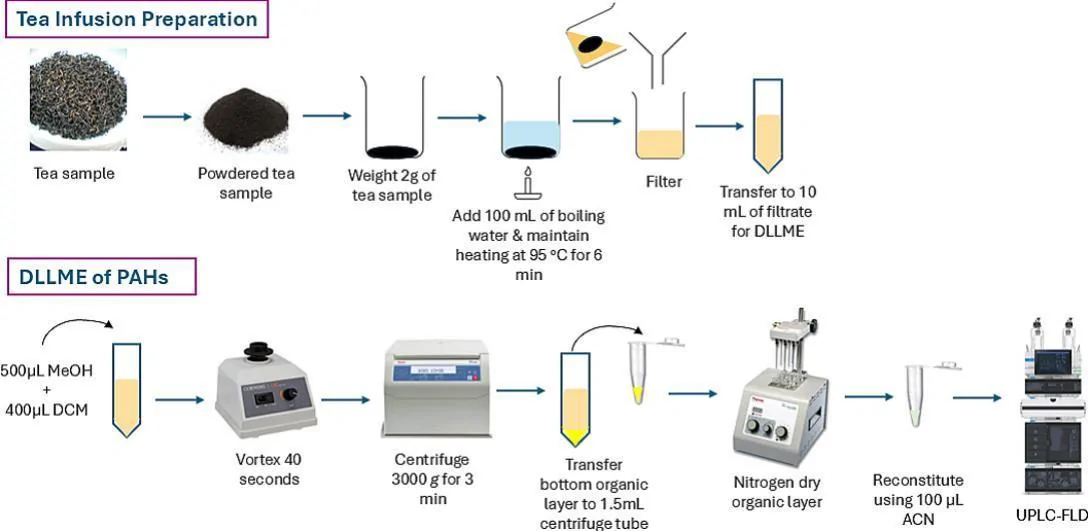

## 1 Introduction

Tea, produced from *Camellia sinensis* and *Camellia assamica*, is one of the most widely consumed beverages globally after water (Statista Research Department, 2024). It is commonly classified into four main categories (non-fermented, semi-fermented, fully fermented, and microbial fermented teas), while herbal infusions are generally referred to as tisanes (ISO 20715:2023, 2023). In 2023, global tea consumption was estimated at ∼7.3 billion kg, and is projected to reach 8.3 billion kg by 2029 (Statista Research Department, 2024). Tea consumption has been associated with various health benefits, including antioxidant activity, cardiovascular protection, and cognitive enhancement (Shang et al., 2021). However, recent studies have reported the presence of contaminants in tea leaves, including pesticides, heavy metals, and polycyclic aromatic hydrocarbons (PAHs) (Abd El-Aty et al., 2014).

PAHs are organic compounds consisting of two or more fused aromatic rings (Peeters & Cami, 2020). They are mainly formed through the incomplete combustion of organic matter, which may occur naturally during forest fires and volcanic eruptions (Patel et al., 2020). Anthropogenic activities, including biomass burning coke production, vehicular emissions and waste incineration, are also major contributors to PAH formation (Patel et al., 2020). In food, PAHs can be formed during high-temperature processing methods such as baking, frying, grilling, and smoking (Paris et al., 2018). Plant derived foods, including tea leaves, may accumulate PAHs from soil, air or water during cultivation (Li, Xue*,* et al., 2025; Lin et al., 2006). PAH contamination may also occur during tea manufacturing, particularly during processing steps involving high temperature (Chiang et al., 2021; Grover et al., 2013).

Long-term exposure to PAHs has been associated with harmful health effects, including carcinogenicity, genetic damage, and immune system impairment (Sun, Song*,* et al., 2021). The United States Environmental Protection Agency (U.S. EPA) has identified 16 PAHs as priority pollutants due to their potential health risks. These include benz(*a*)anthracene (BaA), benzo(*a*)pyrene (BaP), benzo(*b*)fluoranthene (BbFA), benzo(*k*)fluoranthene (BkFA), chrysene (CHR), dibenz(*a*,*h*)anthracene (DBahA), indeno(1,2,3-c,d)pyrene (IP), acenaphthene (ACE), fluorene (FLU), phenanthrene (PHE), anthracene (ANT), benzo(*ghi*)perylene (BghiP), pyrene (PYR), naphthalene (NAP), acenaphthylene (ACY), and fluoranthene (FLT) (United States Environmental Protection Agency, 2014). Of these, eight PAHs (BaP, BaA, BbFA, BkFA, BghiP, CHR, DBahA, and IP) have been identified and recommended by the European Food Safety Authority (EFSA) (European Food Safety Authority, 2008).

The extraction of PAHs from food matrices remains challenging due to matrix interferences and trace level concentrations at which these compounds are often present. Liquid-liquid extraction (LLE) (Trouvé et al., 2022) and QuECHERS (Tfouni et al., 2018) are widely used liquid-based extraction methods for PAHs. However, both methods typically require large sample and solvent volumes and involve laborious procedures. Solid phase extraction (SPE), including dispersive SPE (Azizi et al., 2020), dispersive-micro SPE (Shi et al., 2019), magnetic SPE (Shi et al., 2016), and micro-SPE (Boon et al., 2019) have been other promising options reported for PAHs extraction. However, conventional SPE cartridges are relatively costly and generally not reusable (Notardonato et al., 2021). Therefore, there is a need for the development of a rapid, simple, and solvent efficient extraction method for PAHs analysis in complex food matrices.

Dispersive liquid–liquid microextraction (DLLME) is a ternary solvent system consisting of sample solution, extraction solvent, and dispersive solvent (Sajid, 2022). This technique is relatively simple to adopt and requires only microliter volumes of solvent, making it a more environmentally favorable alternative to conventional extraction methods (Rivera-Vera et al., 2019; Yan & Wang, 2013). DLLME has been reported to exhibit higher greenness scores (AGREE and AGREEprep) than LLE and SPE for the extraction of targeted analytes in complex food matrices (Al Wade et al., 2025; Clivillé-Cabré et al., 2025). A recent study by Rivera-Vera et al. (2019) demonstrated the applicability of ionic liquid-based DLLME for extracting six PAHs (BaA, CHR, BbFA, BkFA, BaP, and DBahA) from tea matrices, followed by high-performance liquid chromatography coupled with fluorescence detection (HPLC-FLD) analysis. Similarly, Mesías-Salazar et al. (2023) reported the extraction of the same six PAHs using hydrophobic deep eutectic solvent-based DLLME coupled with ultra high-performance liquid chromatography coupled with fluorescence detection (UHPLC-FLD). However, both methods require additional solvent preparation steps for the ionic liquid or deep eutectic solvent, which may limit their practicality for routine laboratory control. Gas chromatography-mass spectrometry (GC-MS) is widely used for routine detection of PAHs in environmental and food matrices due to its high sensitivity and regulatory compliance (ISO 18287., 2006; United States Environmental Protection Agency, 1984). While the use of GC-MS/MS is also expanding because of its enhanced capability for detecting trace levels of alkylated, nitro, and oxy-PAHs (Mueller et al., 2019; Zhang et al., 2022). Nevertheless, HPLC–FLD remains a commonly used alternative for PAHs quantification due to its simplicity, selectivity, and sensitivity (Chiang et al., 2020; United States Environmental Protection Agency, 1986). In addition, UPLC which uses smaller particle size (<2 μm) columns, provides sharper peaks and higher separation efficiency is gaining popularity for PAHs analysis (Culqui et al., 2026; Silva et al., 2017).

In addition to quantifying contamination levels, health risk assessment is essential for evaluating the potential health implications associated with dietary exposure to chemical contaminants through food consumption. Previous studies on PAHs in food and beverages have highlighted the importance of dietary risk assessment for evaluating potential consumer health risks associated with PAH exposure (Khalili et al., 2023; Moazzen et al., 2022; Naghashan et al., 2023; Shariatifar et al., 2024; Sharifiarab et al., 2022). Among the available risk characterization approaches, the margin of exposure (MOE) method is widely used for genotoxic and carcinogenic contaminants, by comparing estimated dietary exposure with a toxicological reference value (Busk, 2010; Dorne et al., 2009). Given the genotoxic and carcinogenic properties of PAHs, EFSA recommends the MOE approach for evaluating potential risks associated with dietary exposure to these compounds (European Food Safety Authority, 2008).

In Malaysia, annual tea consumption reached 40,000 t in 2022, with an average per capita consumption of 1.19 kg (World Population Review, 2025). Despite increasing concern over PAHs contamination and potential exposure to PAHs through tea consumption, no studies have yet reported the levels of EFSA-recommended PAHs in teas and tisanes available in the Malaysian market. Previous studies have typically focused on specific tea types, a limited number of PAH analytes (Pincemaille et al., 2014), or tea products from other geographical regions (Ma et al., 2023), making it challenging to evaluate contamination patterns and potential exposure risks relevant to Malaysian consumers. Therefore, this study aims to develop and validate a DLLME-UHPLC-FLD method for the simultaneous determination of the eight EFSA-recommended PAHs (BaP, BaA, BbFA, BkFA, BghiP, CHR, DBahA, and IP) in tea products marketed in Malaysia. The proposed method offers improved analytical sensitivity, rapid analysis, low cost, and good greenness performance (AGREE assessment) supporting its potential application in commercial laboratories for routine PAHs determination in tea. The PAHs concentrations measured in tea samples were subsequently used to estimate dietary exposure and assess potential health risks among Malaysian consumers through tea consumption. In addition to method development, this study provides the first occurrence data for EFSA-recommended PAHs in teas and tisanes marketed in Malaysia. These findings provide evidence-based baseline information that may support food safety authorities in future monitoring, consumer exposure evaluation, and risk-based decision-making related to PAHs contamination in tea products.

## 2 Materials and methods

### 2.1 Chemicals and reagents

BaP, BaA, BbFA, BghiP, CHR, DBahA, and IP were purchased from Agilent Technologies (Santa Clara, California). BkFA was purchased from Sigma-Aldrich (St. Louis, Missouri). HPLC grade acetonitrile (ACN) and methanol (MeOH) were obtained from Fisher Scientific (Waltham, Massachusetts). Carbon tetrachloride, chloroform, and dichloromethane (DCM) were purchased from Merck (Darmstadt, Germany). Hexane was purchased from QREC (Asia) Sdn. Bhd. (Selangor, Malaysia). Ultrapure water (UPW) was obtained from Barnstead Smart2Pure water purification system from Thermo Scientific (Waltham, Massachusetts).

### 2.2 Tea samples and tea infusion preparation

Forty-three tea samples (17 fully fermented teas, 6 semi-fermented teas, 6 non-fermented teas, 6 post-fermented teas, and 8 tisanes) were purchased from local supermarkets and stores located in Penang and Kedah. All teas were powdered using commercially available electric grinder, stored in airtight containers, and kept at room temperature (25 °C), protected from direct light, until analysis. The origin and types of teas are detailed in Table S1 (Supplementary Information). 2.0 g of dried tea leaf was weighed and immersed in 100 mL of UPW at 95 °C for 6 min, and filtered through Whatman 1, 110 mm diameter, 11 μm particle retention filtered paper (Cytiva, Marlborough, Massachusetts) (ISO 3103, 2019; Mañana-López et al., 2021). The collected tea infusions were rest to room temperature and 10 mL were used as a working sample for the DLLME process as described in Section 2.4.

### 2.3 Standard solutions

Individual PAHs with an initial concentration of 100 mg L^−1^ were diluted using acetonitrile to obtain primary stock solutions of 20 mg L^−1^. Standard mixtures of the eight PAHs (1 mg L^−1^) were then prepared by diluting the primary stock solutions with acetonitrile. All primary stock solutions and standard mixtures are stored in an amber vial at 4 °C until used.

### 2.4 DLLME procedure

10 mL of tea infusion was taken and placed in a 15 mL centrifuge tube. Then, 500 μL of MeOH was added to the tea infusion, followed by rapid injection of 400 μL of DCM. The tube was then covered, vortex-agitated for 40 s, and centrifuged at 3000 ×*g* for 3 min. Subsequently, the aqueous phase on top is discarded and the bottom extraction solvent was carefully withdrawn into a new microcentrifuge tube. Then, 100 μL of the extraction was taken and evaporated to dryness under a gentle nitrogen stream. The resulting residue was reconstituted in 100 μL ACN and filtered through a 0.22 μm nylon membrane filter (Agilent Technologies, Santa Clara, California). The filtrate was then stored in a HPLC amber glass vial prior to UPLC-FLD analysis. A schematic representation of the DLLME procedure is depicted in Fig. S1 (Supplementary Information).

### 2.5 UPLC-FLD system

Separations were conducted on an Agilent 1290 ultra performance liquid chromatographic system (UPLC) (Agilent Technologies, Santa Clara, California) equipped with a quaternary pump with integrated column compartment (G7104A), an autosampler (G7129B), and a fluorescence detector (FLD) (G7121B). Chromatographic separation was effected using Poroshell 120 EC-C18 column (2.1 × 100 mm, 1.9 μm particle size) at column temperature of 35 °C. The initial flow rate was set at 0.45 mL/min for 1 min and ramped to 0.75 mL/min over 9 min and maintained for 9 min. The flow rate was then returned to 0.45 mL/min and held for 8 min. Gradient elution was performed with UPW (A) and ACN (B). The initial gradient was 60% B for 1 min, and 63.6% B at 10 min, 64.8% B at 13 min, 100% B at 14 min and maintained for 5 min, returned to 60% B at 20 min and held for 8 min. The injection volume was 5 μL, and fluorescence detection was performed at an excitation wavelength of 270 nm with 4 emission wavelengths: 385 nm (CHR), 405 nm (BaA and DBahA), 420 nm (BbFA, BkFA, BaP, BghiP), and 510 nm (IP).

### 2.6 Data handling and statistical analysis

Agilent OpenLab CDS version 2.7 (Agilent Technologies, Santa Clara, California) was used for data acquisition and the obtained peak resolution was calculated with the half-width method as follows:

$$\mathrm{Rs}=1.18\times \frac{t_{R2}-t_{R1}}{W_{50\left(1\right)}+W_{50\left(2\right)}}$$

Where t_R2_ and t_R1_ are retention times of the first and second analytes, W_50_ is the peak width at half height in min (USP, 2022). Original data acquired from OpenLab CDS was exported to CSV format, and Minitab version 21.4.2 (Minitab, State College, Pennsylvania) was used for the reconstruction of chromatograms. All experiments were conducted in triplicate, and the obtained data were presented as mean ± standard deviation. Studies of the correlation coefficient and linear regression, assessment of repeatability, recovery, mean, standard deviation and % RSD were performed using Microsoft Excel 365 (Microsoft, Redmond, Washington). Calibration curves were constructed using least-squares linear regression of peak area response against analyte concentrations, and linearity was evaluated using the coefficient of determination (R^2^). The limit of detection (LOD) and limit of quantification (LOQ) were statistically determined using the standard deviation of the response and the slope of the calibration curve using the following equations:

$$\mathrm{LOD}=\frac{3.3\sigma }{S}$$

$$\mathrm{LOQ}=\frac{10\sigma }{S}$$

where σ is the standard deviation of the response and S is the slope of the regression line (ICH Harmonised Guideline, 2022).

### 2.7 Method validation

The applicability of the UPLC-FLD method was evaluated in terms of linearity, LOD, LOQ, precision (inter- and intra- day), and accuracy based on the guidelines defined by the International Council for Harmonization (ICH Harmonised Guideline, 2022). Several tea samples were screened to identify a blank matrix with non-detectable PAHs levels. As no completely PAH-free sample was found, the tea sample with the lowest PAH content was selected as the matrix blank and spiked with known amounts of PAHs to prepare matrix-matched calibration standards. Matrix-matched calibration curves were established by plotting peak areas against concentrations at five levels (75, 100, 200, 300, 400 ng L^−1^) for CHR, BaA, BbFA, BkFA, BaP, DBahA, and BghiP. Meanwhile, the calibration curve for IP was constructed using concentrations of 200, 300, 400, 500, 1000 ng L^−1^. The LOD and LOQ were estimated by performing 7 procedural blanks (3 injections each) using the developed DLLME-UPLC-FLD method by multiplying the standard deviation of the background response by 3.3 and 10 times the slope. Intraday precision was assessed on the same day with nine replicates (*n* = 9), while interday precision was evaluated over three consecutive days with a total of 27 replicates (*n* = 27). Precision and accuracy of the developed method were examined at three concentration levels, 85 ng L^−1^, 250 ng L^−1^ and 320 ng L^−1^ for CHR, BaA, BbFA, BkFA, BaP, DBahA, and BghiP, and 250 ng L^−1^, 320 ng L^−1^ and 850 ng L^−1^ for IP. The results for precision (intra- and inter- day) were expressed as relative standard deviation (RSD) for retention time and peak area. Meanwhile, the % recovery was determined using the following equation:

$$\mathrm{Recovery}\;\left(\%\right)=\frac{\mathrm{Measured concentration for spiked sample}}{\mathrm{Spiked concentration}}\times 100$$

The enrichment factor (EF) was calculated using the following formula:

$$\mathrm{EF}=\frac{C_{\mathrm{sed}}}{C_{0}}$$

where C_sed_ is the concentration of PAHs in the extraction solvent after DLLME and C_0_ is the initial concentration of analytes in aqueous phase.

### 2.8 Greenness evaluation

The Analytical GREEnness (AGREE) calculator, which is based on the 12 principles of Green Analytical Chemistry (GAC), was used to evaluate the greenness of the developed analytical method (Pena-Pereira et al., 2020). Sample preparation procedure, which is often the most resource-intensive and hazardous stage of the analytical workflow was evaluated separately using AGREEprep, a metric based on the 10 principles of Green Sample Preparation (GSP) (Wojnowski et al., 2022).

### 2.9 Deterministic dietary exposure and margin of exposure

The estimated daily intake (EDI, mg/kg BW per day) of PAHs through tea consumption among Malaysian adult was calculated using a deterministic approach as equation below (World Health Organization, 2020):

$$\mathrm{EDI}=\frac{C\times \mathrm{IR}}{\mathrm{BW}}$$

where C (ng kg^− 1^) represents the arithmetic mean concentration of PAHs in each category of tea; IR is the estimated average daily tea consumption rate for Malaysian adult (8.2 g/person/day) (Tan et al., 2024); BW (kg) denotes the average adult body weight (70 kg) (EFSA Scientific Committee, 2012).

To evaluate the potential health risks, the margin of exposure (MOE) for BaP, PAH2, PAH4 and PAH8 were calculated using the benchmark dose lower confidence limit for a 10% response (BMDL_10_) which reflects the lowest dose with 95% certainty at which a 10% increase in tumor incidence may be observed, as proposed by the European Food Safety Authority (EFSA). The MOE was computed using the following formula (European Food Safety Authority, 2005):

$$\mathrm{MOE}=\frac{{\mathrm{BMDL}}_{10}}{\mathrm{EDI}}$$

where BMDL_10_ of BaP is 0.07 mg/kg BW/day, PAH2 is 0.17 mg/kg BW/day, PAH4 is 0.34 mg/kg BW/day, and PAH8 is 0.49 mg/kg BW/day (European Food Safety Authority, 2008).

## 3 Result & discussions

### 3.1 Optimization of UPLC-FLD conditions

In this study, UPLC-FLD parameters, including column type, initial mobile phase composition, flow rate, column temperature, injection volume, excitation and emission wavelengths were systematically optimized.

#### 3.1.1 Optimization of chromatographic separation parameters

Preliminary experiments were conducted to evaluate the chromatographic separation of PAHs using Poroshell 120 PFP (2.1 × 100 mm, 1.9 μm) and Poroshell 120 EC-C18 (2.1 × 100 mm, 1.9 μm). The PFP column is known for its unique selectivity toward aromatic compounds, amino-PAHs, and monohydroxylated-PAHs (Li, Cui*,* et al., 2025; Martinefski et al., 2019). However, baseline separation of the targeted PAHs was not achieved with adjustment of chromatographic parameters with co-elutions occurring for BbFA and BkFA (Fig. S2; Supplementary Information). Several recent studies have demonstrated the applicability of using C18 columns for the separation of PAHs (Singh et al., 2022; Sun, Han*,* et al., 2021). In particular, the introduction of core-shell particles has substantially reduced band broadening caused by multiple inter-particle pathways and longitudinal diffusion, enabling highly efficient and rapid chromatographic separations (González-Ruiz et al., 2015). Therefore, Poroshell 120 EC-C18 was selected for subsequent method optimization. The mobile phase composition has been known to play a major influence on compound separation and peak shape (Yuan et al., 2025). Initial mobile phase tested was ACN:UPW (70:30, v/v) reported by Sun, Han, et al. (2021) for analysis of PAHs in honey. Under these conditions, BaA was not adequately separated from CHR, with a resolution factor (Rs) of 0.72. To improve chromatographic separation, different ACN:UPW ratios (65:35, 60:40, 55:45, and 50:50, v/v) were then evaluated. Reducing the ACN proportion improved the resolution of BaA. However, a higher water proportion resulted in longer retention times. Therefore, ACN:UPW at 60:40 (v/v) was selected as the optimal initial mobile-phase composition condition. The effect of flow rate on backpressure, chromatographic resolution and retention time was systematically investigated. Experiments were conducted at flow rates ranging from 0.20 to 0.50 mL/min, and 0.45 mL/min provided the best peak resolution while maintaining an acceptable analysis time (∼19 min). To further shorten the total run time without compromising separation, the flow rate was gradually increased from 0.45 mL/min (backpressure of ∼670 bar) to 0.8 mL/min (backpressure of ∼1108 bar) after the elution of CHR and BaA. As expected, increasing the flow rate increased the column back pressure. Nevertheless, the Rs remained greater than 1.0, indicating acceptable separation. Thus, the flow rate ramp from 0.45 to 0.75 mL/min that provided the best overall separation, analysis time, and acceptable back pressure (∼1044 bar) were used for subsequent study. The effect of column temperature on chromatographic performance was evaluated from 25 to 45 °C in 5 °C increment. Increasing the column temperature can reduce mobile phase viscosity, improve mass transfer efficiency, and lower column back pressure (Roehrer & Minceva, 2019). However, results indicated that higher temperature led to a loss of peak resolution. Therefore, 35 °C was selected as the optimal operating column temperature, as it shortened total analysis time to less than 12 min (reduction of 36.8%), lowered the back pressure from 1044 bar to 937.5 bar, and still provided acceptable peak resolution. The injection volume was briefly evaluated to achieve sufficient sensitivity without compromising peak shape and resolution. Injection volumes ranging from 2 μL to 6 μL were investigated. An injection volume of 5 μL provided consistent and reproducible results, with minimal sample overload and acceptable signal intensity for all analytes. Hence, 5 μL was selected for subsequent study.

#### 3.1.2 Selection of excitation and emission wavelengths

Fluorescence detection is strongly influenced by the choice of excitation and emission wavelengths, and wavelength optimization can substantially improve detection sensitivity for fluorophoric compounds. The optimal emission wavelength for each PAH was first determined by fixing the excitation wavelength at 230 nm, and scanning the emission range from 280 nm to 1200 nm. Results indicated that each PAH exhibited a distinct optimal emission wavelength: CHR at 384 nm, BaA at 402 nm, BbFA at 454 nm, BkFA at 427 nm, BaP at 416 nm, DBahA at 408 nm, BghiP at 424 nm, and IP at 508 nm (Fig. S3). Subsequently, the optimal excitation wavelengths were then determined by fixing the emission wavelength for each PAH and scanning the excitation range from 200 nm to 1200 nm. The resulting excitation maxima were 272 nm, 281 nm, 263 nm, 235 nm, 385 nm, 299 nm, and 256 nm, respectively. These results indicated that the selected excitation wavelength must balance sensitivity across all target analytes. Time programmed wavelength switching is commonly used in PAHs determination using HPLC-FLD with different excitation/emission wavelength pairs applied at specific retention-time windows (Chiang et al., 2021; Tfouni et al., 2018). Nonetheless, BghiP and IP were eluted closely (Rs of 0.22), and conventional time-programmed wavelength switching was found to cause peak distortion. Therefore, multi-wavelength FLD detection was employed in this study. A single excitation wavelength of 270 nm was selected, while four emission wavelengths were monitored simultaneously: 385 nm for CHR, 510 nm for IP, 405 nm for BaA and DBahA, and 420 nm for BbFA, BkFA, BaP, and BghiP. Fig. S4 shows the chromatograms of the PAHs separated using the optimized conditions (Table S2). The developed method achieved separation of the eight PAHs in <12 min, which is shorter than reported HPLC-FLD method (15 min for 6 PAHs) (Rivera-Vera et al., 2019) and is comparable to GC-MS method (13 min for 8 PAHs) (Huynh et al., 2024). Nevertheless, the proposed method is longer compared to ultra-high performance liquid chromatography-atmospheric pressure photoionization-tandem mass spectrometry (UHPLC-APPI-MS/MS) method for the determination of 20 PAHs within 15 min (Lung & Liu, 2015).

### 3.2 Optimization of DLLME conditions

To achieve optimal extraction efficiency, key parameters affecting DLLME performance, including the type and volume of extraction and disperser solvent, salt content, pH, sample volume, vortex duration, and centrifugation speed and time, were systematically investigated.

#### 3.2.1 Selection of extraction and disperser solvent

**Fig. 1:**
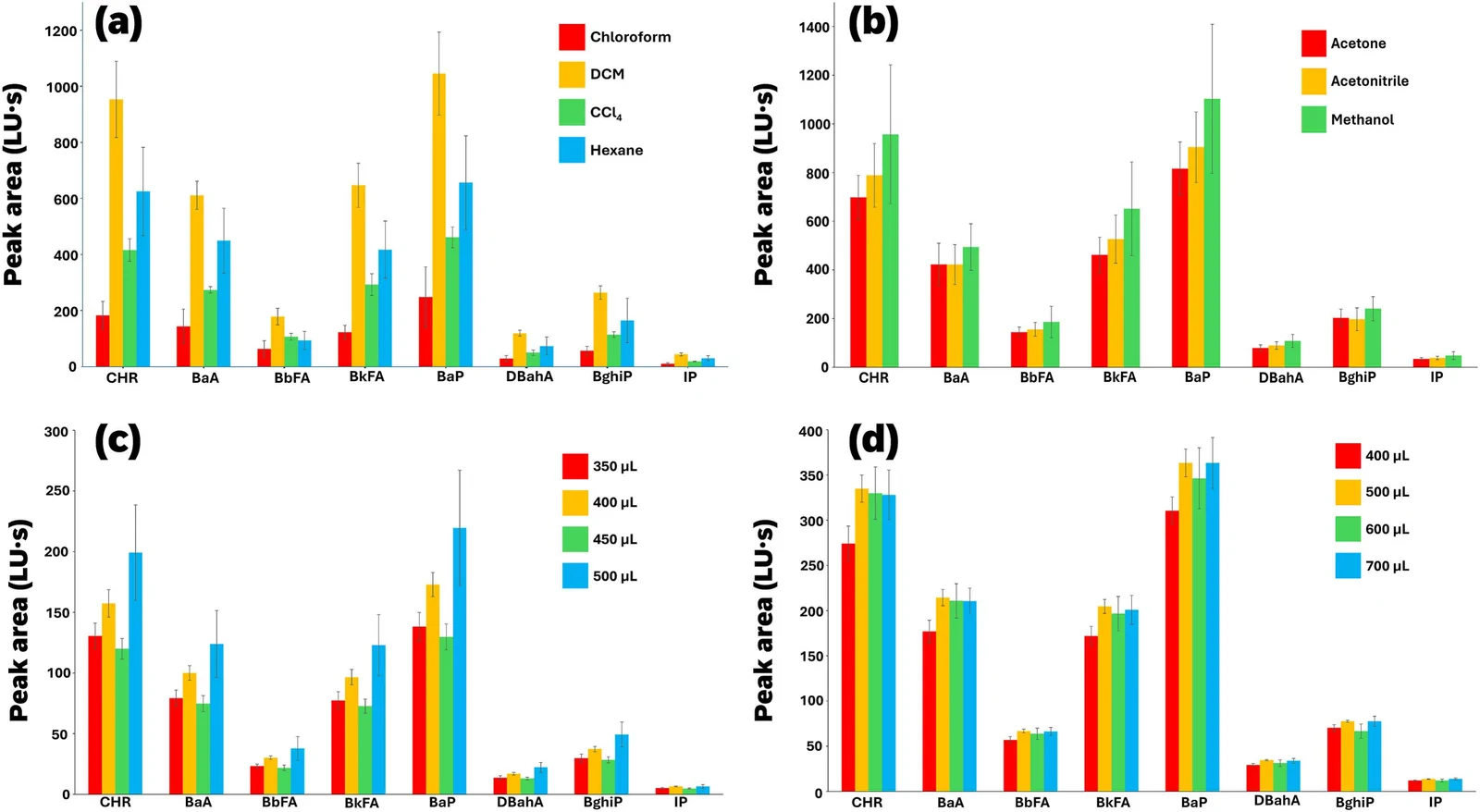
Effects of (a) extraction solvent type, (b) disperser solvent type, (c) DCM volume, and (d) MeOH volume on the extraction efficiency of PAHs. Error bars represent the standard deviation (*n* = 3).

The selection of extraction solvents is important for improving extraction efficiency and selectivity. Various solvents, including hexane (Guo et al., 2022), DCM (Lin et al., 2005), and ethyl acetate (Shinde et al., 2025) have been used for PAHs extraction in liquid-liquid and solid-liquid extraction methods. In DLLME, the extraction solvent should effectively extract the target PAHs, exhibit low solubility in water, and miscible with dispersive solvent (Rezaee et al., 2006). In this study, chloroform (polarity: 4.1) (Ricciutelli et al., 2006), DCM (polarity: 3.1) (Ricciutelli et al., 2006), carbon tetrachloride (polarity: 1.7) (Rashid, 2008), and *n*-hexane (polarity: 0) (Ricciutelli et al., 2006), were evaluated initially with acetone used as the dispersive solvent. Among the extraction solvents tested, DCM provided the highest extraction efficiency for the target PAHs (Fig. 1a). This can be attributed to the moderate polarity and high polarizability of DCM, which facilitates strong dispersive and dipole-induced interactions with the extensive π-electron clouds of PAHs. The hydrophobic character and polarizable chlorine atoms in DCM may also promote dispersive and dipole-induced interactions with the delocalized π-electrons of fused aromatic rings, which collectively favour portioning of PAHs from the aqueous phase into DCM DLLME (Doveiko et al., 2024; Kim et al., 2026). In addition, DCM is generally associated with lower environmental and chronic health impacts than chloroform, carbon tetrachloride and hexane (Kokosa, 2019). Therefore, DCM was selected as the extraction solvent for subsequent studies. Acetone, ACN, and MeOH were then evaluated for their suitability as dispersive solvent. Results (Fig. 1b) showed that MeOH provided the highest recovery when paired with DCM (as extractant). This phenomenon could be due to the nature of methanol, which is highly polar and miscible with both phases, resulting in the least interference with the partitioning of PAHs into the DCM phase (Vera-Avila et al., 2013). Therefore, DCM/MeOH solvent was used for further optimization.

#### 3.2.2 Effect of extraction and dispersive solvents volume

The effect of extraction solvent volume on the extraction recovery was evaluated using DCM volumes ranging from 350 to 500 μL, while the dispersive solvent (MeOH) volume was kept constant at 600 μL. Fig. 1c showed that 500 μL of DCM resulted in the highest extraction recovery for all PAHs. However, greater variability was observed at this volume, as indicated by the larger standard error bars (RSD > 17%). This suggests that larger extraction solvent volume improved solute solubilization and mass transfer, but may compromise reproducibility due to inconsistencies in droplet formation during phase separation (Teshale & Taye, 2019). Therefore, 400 μL of DCM was selected as the optimal volume, providing good extraction efficiency and reproducibility. The effect of dispersive solvent volume was then evaluated using MeOH volumes ranging from 400 to 700 μL, while the DCM volume fixed at 400 μL. Fig. 1d showed that 500 μL of MeOH provided the highest extraction recovery. Interestingly, extraction efficiency was reduced when MeOH volume was lower than 500 μL. This might be attributed to insufficient dispersion that limited the formation of fine extraction solvent droplets (Teshale & Taye, 2019). On the other hand, MeOH volume >500 μL increased partial solubilization of the extractant in the aqueous phase, thereby reducing the sedimentation phase volume and compromising extraction repeatability (Zaid et al., 2025). Thus, 500 μL of MeOH was selected as the optimal dispersive solvent.

#### 3.2.3 Effect of sample salt content and pH

**Fig. 2:**
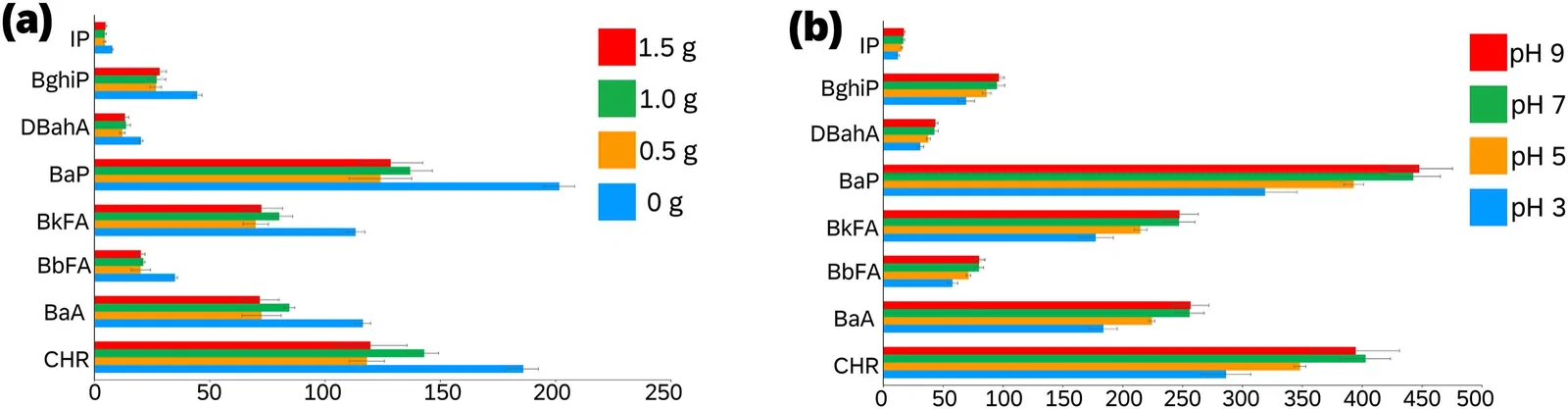
Effects of (a) salt content and (b) sample pH on the extraction efficiency of PAHs. Error bars represent the standard deviation (*n* = 3).

The addition of salt is commonly used in DLLME to modify the ionic strength of the aqueous phase, promote phase separation, and enhance extraction efficiency (Raynie, 2023). To assess its effects, different amounts of sodium chloride (NaCl), ranging from 0 to 1.5 g, were added to the aqueous sample solution. The results (Fig. 2a) showed that the highest extraction performance was achieved in the absence of salt. This may be attributed to the non-polar nature of the target PAHs, and salt addition did not typically enhance their partitioning into the extraction solvent (Ma et al., 2022). Additionally, salt addition can increases the viscosity of the aqueous phase, thereby slowing analyte diffusion and reducing mass-transfer efficiency from the aqueous phase to the organic solvent (Zhu et al., 2026). This observation is consistent with previous studies reporting a negative effect of salt addition on the extraction of PAHs (Li et al., 2019; Zhou et al., 2018). Therefore, no salt was added for subsequent DLLME experiments. The effect of sample pH on the extraction efficiency was studied at pH 3, 5, 7, and 11. As shown in Fig. 2b, the extraction efficiency increased from pH 3 to pH 7 and remained relatively constant at pH 9. This observation might be explained by the non-ionizable nature of PAHs which do not undergo acid–base dissociation (Masemola et al., 2025). Thus, pH was not expected to strongly affect their extraction behavior. Therefore, the neutral condition (pH 7), which provided better and more consistent recovery was selected for subsequent experiments.

#### 3.2.4 Effect of sample volume, vortex duration, centrifugation time, and speed

Sample volume is an important factor in DLLME because it influences analytes partitioning and enrichment. Sample volumes of 6, 8, 10, and 12 mL, with concentration of 5 ng L^−1^, were evaluated. Results (Fig. S5a) showed that extraction performance of PAHs increased up to 10 mL and decreased slightly at 12 mL. The lower recovery at higher sample volumes may be due to reduced partitioning efficiency, which decreases the amount of analytes transferred into the extraction solvent (Mesías-Salazar et al., 2023). Therefore, 10 mL was selected for further studies. Vortexing enhances mass transfer by promoting the dispersion of the extraction solvent into fine droplets, thereby increasing the contact area between the aqueous and the organic phases (Mesías-Salazar et al., 2023; Rivera-Vera et al., 2019). As extraction efficiency is strongly influenced by vortex durations, vortex times of 20, 40, and 60 s were evaluated. As shown in Fig. S5b, extraction efficiency increased from 20 s to 40 s, while only a marginal improvement was observed at 60 s. Hence, 40 s was selected as the optimal vortex duration. Centrifugation is a crucial step in the DLLME, as it facilitates the disruption of the emulsion formed during vortexing and enhances phase separation (Mesías-Salazar et al., 2023; Tolcha & Megersa, 2018). Therefore, centrifugation speed and duration were systematically evaluated. Centrifugation speeds ranging from 1500 to 3000 ×*g* and centrifugation times of 1, 3, 5 and 7 min were tested. Results (Fig. S5c) show that extraction performance improved with increasing centrifugation speed. However, speeds above 3000 ×g were not investigated due to instrumental limitation. Therefore, 3000 ×*g* was selected. Centrifugation time was then optimized under previously selected experimental conditions. As shown in Fig. S5d, a centrifugation time of 3 min provided the best extraction efficiency. Longer centrifugation times did not substantially improve recovery. Therefore, 3 min was selected as the optimal centrifugation time.

### 3.3 Analytical performance of the DLLME–UPLC-FLD method

The developed DLLME-UPLC-FLD method was validated by determining the linearity, LOD, LOQ, precision, and recovery (Table 1). Matrix-matched calibration curves were constructed using five concentrations levels over the ranges of 200–1000 ng L^−1^ for IP and 75–400 ng L^−1^ for the other PAHs. A higher concentration range was used for IP calibration because of its low fluorescence quantum yield and relatively short fluorescence lifetime that resulted in higher detection limits (Pandey et al., 2017; Saxena et al., 1980; Zhang et al., 2020). Good linearity was obtained with correlation coefficients (R^2^) > 0.99. The LOD and LOQ were determined as three and ten times the standard deviation of the blank tea sample, respectively. LODs and LOQs range from 3.87 to 32.5 ng L^−1^ and from 11.73 to 98.47 ng L^−1^, respectively. The developed method demonstrated lower LOD values than those reported by Azari et al. (2023) (9–60 ng L^−1^) for the determination of 16 PAHs in soft drinks and non-alcoholic beers using magnetic solid-phase extraction and gas chromatography-mass spectrometry (GC-MS) and comparable sensitivity to the membrane-assisted solvent extraction combined with GC-MS method (3–40 ng L^−1^) for the determination of 16 PAHs in river water, apple juice, red wine and milk (Rodil et al., 2007). Intra-day (*n* = 9) precision, inter-day (*n* = 27) precision, and accuracy were evaluated by spiking PAH standards at three concentration levels (250, 320, 850 ng L^−1^ for IP, and 85, 250, 320 ng L^−1^ for the other PAHs). The selected spiking concentrations were set above the LOQs and within the calibration range, corresponding to low, medium, and high concentration levels. Intra-day precision was assessed using 9 replicates on the same day, while inter-day precision was evaluated using 27 measurements over three consecutive days. The relative standard deviations (RSDs) for intra-day retention times and peak areas were ≤0.10% and 9.21%, respectively. For inter-day precision, the RSDs for retention times and peak areas were ≤1.60% and 9.91%, respectively. The method also achieved good recoveries of 85.13–101.71%, with standard deviations <9.3% for all studied PAHs. These values are within the acceptable recovery range (80% - 120%) recommended by the ICH guideline (ICH Harmonised Guideline, 2022). Additionally, these values are comparable to those previously reported by Guo et al. (2022) method (82.1–97.4%) for analysis of 22 PAHs in Chinese tea using multi-walled carbon nanotubes continuous solid-phase extraction (MWCNTs-CSPE) combined with high-performance liquid chromatography with ultraviolet and fluorescence dual detectors (HPLC-DAD-FLD). The method also provided enrichment factors ranging from 35 to 48 (Table 1), confirming its applicability for PAHs determination in tea matrices. The analytical performance of the validated DLLME-UPLC-FLD method and previously reported LC-based methods for the determination of PAHs in tea samples is summarized in Table S3 (Supplementary Information).

**Table 1:** Analytical validation parameters for the studied PAHs determined using the DLLME-UPLC-FLD method.

Polyaromatic Hydrocarbons	Linearity					Intraday precision (% RSD, *n* = 9)				Interday precision (%RSD; *n* = 27)				Mean recovery (% ± SD)				EF
	Regression Equation	Linear Range (ng L^−1^)	R^2^	LOD (ng L^−1^)	LOQ (ng L^−1^)	85 ng L^−1^	250 ng L^−1^	320 ng L^−1^	850 ng L^−1^	85 ng L^−1^	250 ng L^−1^	320 ng L^−1^	850 ng L^−1^	85 ng L^−1^	250 ng L^−1^	320 ng L^−1^	850 ng L^−1^	
CHR	y = 0.043x − 0.1113	75–400	0.9975	7.58	22.98	a3.71 b(0.02)	4.15 (0.07)	1.86 (0.04)	–	2.43 (1.10)	9.91 (1.41)	3.11 (0.97)	–	97.45 ± 2.29	94.43 ± 9.26	90.34 ± 2.79	–	36
BaA	y = 0.0298x + 0.2619	75–400	0.9974	20.95	63.48	2.47 (0.02)	3.19 (0.07)	3.45 (0.03)	–	3.81 (1.09)	9.05 (1.41)	2.59 (0.98)	–	91.08 ± 3.87	98.64 ± 9.25	89.01 ± 2.38	–	35
BbFA	y = 0.0075x − 0.0229	75–400	0.9996	3.87	11.73	1.75 (0.01)	4.68 (0.05)	1.23 (0.02)	–	5.88 (0.92)	8.91 (1.36)	2.61 (0.90)	–	91.02 ± 5.14	97.30 ± 8.56	92.75 ± 2.40	–	38
BkFA	y = 0.0212x − 0.1846	75–400	0.9971	14.22	43.09	3.23 (0.01)	6.58 (0.05)	4.86 (0.02)	–	7.00 (0.98)	5.40 (1.35)	3.77 (0.90)	–	89.28 ± 5.53	88.60 ± 4.60	89.14 ± 3.25	–	38
BaP	y = 0.0474x − 0.1524	75–400	0.9984	7.26	22.01	1.75 (0.01)	4.11 (0.05)	1.67 (0.02)	–	3.32 (1.11)	7.82 (1.27)	3.35 (0.87)	–	89.92 ± 2.86	95.84 ± 7.40	92.00 ± 3.05	–	40
DBahA	y = 0.0039x − 0.1138	75–400	0.9904	23.11	70.04	1.86 (0.05)	9.21 (0.04)	8.0 (0.03)	–	5.23 (0.99)	3.96 (1.60)	6.22 (0.91)	–	88.43 ± 2.83	94.16 ± 3.26	89.30 ± 4.99	–	43
BghiP	y = 0.0114x − 0.2784	75–400	0.9931	4.71	14.26	1.76 (0.04)	2.33 (0.06)	3.21 (0.02)	–	5.22 (0.91)	5.66 (1.54)	2.23 (0.84)	–	85.13 ± 2.94	97.27 ± 4.95	94.98 ± 1.94	–	44
IP	y = 0.0022x − 0.2448	200–1000	0.9936	32.5	98.47	–	2.07 (0.10)	1.16 (0.03)	5.70 (0.07)	–	7.30 (1.43)	3.63 (0.85)	6.45 (0.19)	–	101.71 ± 4.18	97.54 ± 2.28	91.71 ± 5.07	48

a Values are corresponds to the percent relative standard deviation (% RSD) for peak area.

b Values in brackets corresponds to the % RSD for retention time.

### 3.4 Analysis of analytical greenness metrics

**Fig. 3:**
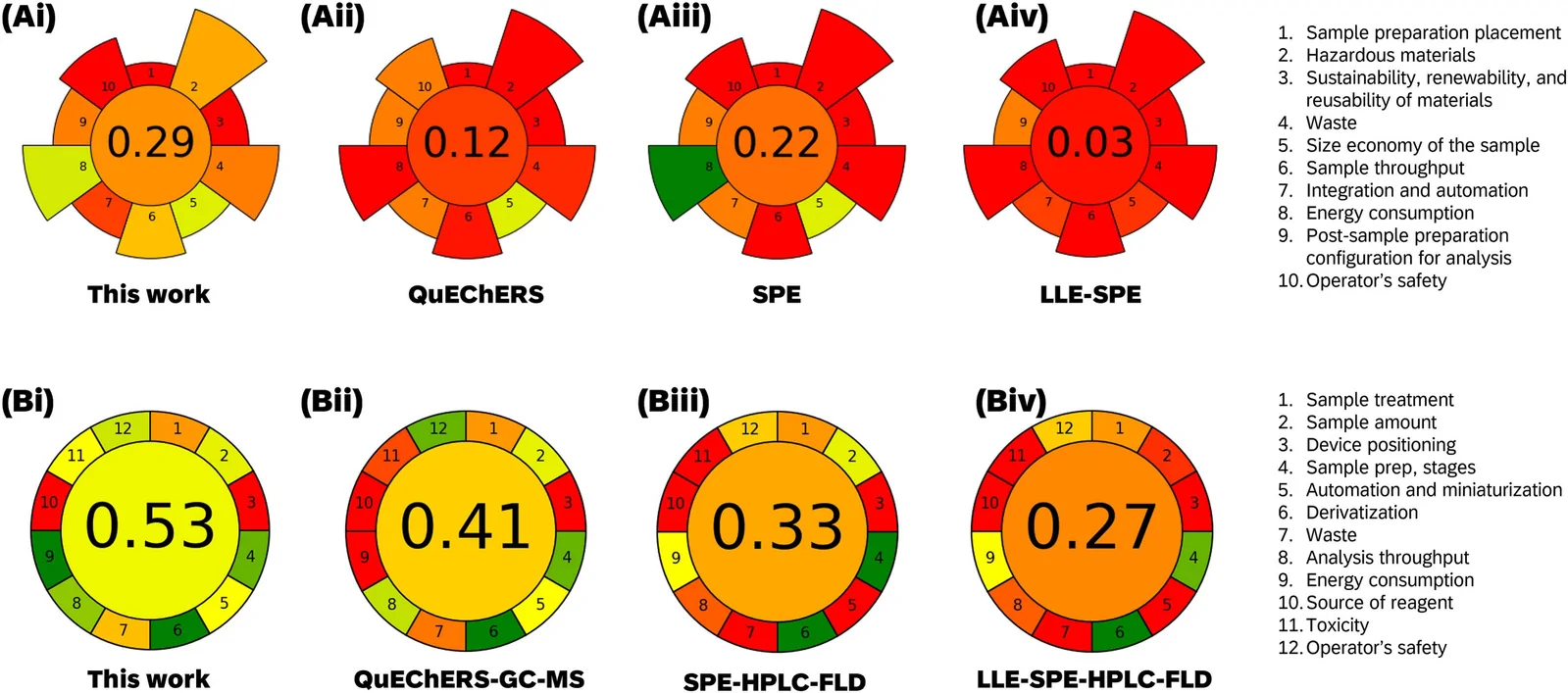
Pictograms generated from AGREEprep and AGREE assessments of procedures for PAH determination in tea: (A) AGREEprep scores for (i) DLLME (this work), (ii) QuEChERS (Jeong et al., 2025), (iii) SPE (Bravo et al., 2019), and (iv) LLE–SPE (Thea et al., 2016); and (B) AGREE scores for (i) DLLME–UPLC–FLD (this work), (ii) QuEChERS–GC–MS (Jeong et al., 2025), (iii) SPE–HPLC–FLD (Bravo et al., 2019), and (iv) LLE–SPE–HPLC–FLD (Thea et al., 2016).

The greenness of the developed DLLME method was assessed using AGREEprep tool and compared with three reported sample preparation techniques for extraction of PAHs in foods and beverages. As shown in Fig. 3(A), the developed DLLME method achieved the highest AGREEprep score (0.29) compared to QuEChERS (0.12) (Jeong et al., 2025), SPE (0.22) (Bravo et al., 2019) and LLE-SPE (0.03) (Thea et al., 2016). This higher score can be attributed to the use of fewer hazardous materials, lower waste generation, shorter extraction time, higher sample throughput, and reduced energy consumption due to the absence of energy-intensive and time-consuming sample preparation steps. However, the DLLME method still has limitations, including manual sample handling and the use of unsustainable and hazardous solvents, particularly DCM. The lower AGREEprep score of QuEChERS compared with SPE may be associated with its extensive use of centrifugation and rotary evaporation, which increased energy consumption. Fig. 3(B) shows the overall AGREE score for the developed DLLME-UPLC-FLD method (0.53) and the reported QuEChERS-GC-MS (0.41) (Jeong et al., 2025), SPE-HPLC-FLD (0.33) (Bravo et al., 2019), and LLE-SPE-HPLC-FLD (0.27) (Thea et al., 2016) methods. The higher AGREE score obtained is mainly attributed to the DLLME workflow, which enables rapid extraction using small volumes of extraction and dispersive solvents. In addition, UPLC reduces energy consumption compared to GC-MS and conventional HPLC, and faster chromatographic separation also improves analytical throughput. Overall, the proposed DLLME-UPLC-FLD method provides a greener and more practical alternative for PAH determination in tea.

### 3.5 Analysis of tea samples using DLLME–UPLC-FLD

**Fig. 4:**
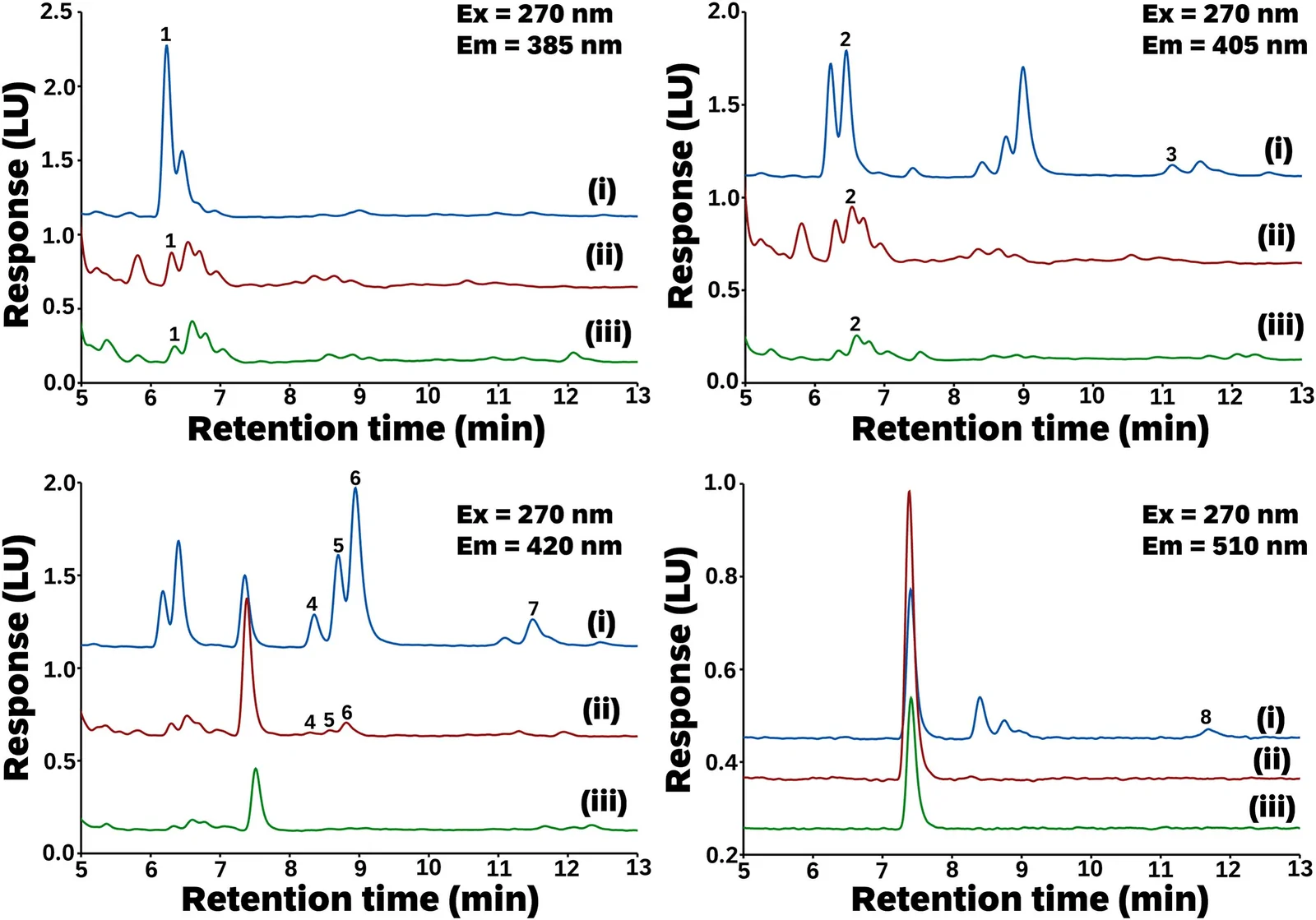
UPLC–FLD chromatograms of (i) sample spiked with PAHs at 250 ppt, (ii) LBT, and (iii) GT 3. Peak assignments: 1, chrysene; 2, benzo(*a*)anthracene; 3, dibenzo(*a*,*h*)anthracene; 4, benzo(*b*)fluoranthene; 5, benzo(*k*)fluoranthene; 6, benzo(*a*)pyrene; 7, benzo(*g*,*h*,*i*)perylene; and 8, indeno(1,2,3-*cd*)pyrene.

The validated DLLME-UPLC-FLD technique was applied to quantify PAHs in 43 tea samples, comprising of 17 fully fermented teas, 6 semi-fermented teas, 6 non-fermented teas, 6 post-fermented teas, and 8 tisanes. Representative chromatograms of selected tea samples are depicted in Fig. 4. The PAHs detected in the tea samples are summarized in Table S1 (Supplementary Information), while the mean, minimum, and maximum concentrations, together with ∑PAH2, ∑PAH4, and ∑PAH8, are detailed in Table 2. Overall, the mean concentration of ∑PAH8 showed the following trend: post fermented tea (144.27 ng kg^−1^) > non fermented tea (31.61 ng kg^−1^) > fully fermented tea (17.10 ng kg^−1^) > semi fermented tea (8.35 ng kg^−1^) > tisanes (4.28 ng kg^−1^).

**Table 2:** Concentrations of PAHs (ng kg−^1^) detected in five categories of tea samples marketed in Malaysia.

	CHR	BaA	BbFA	BkFA	BaP	DBahA	BghiP	IP	∑ PAH 2	∑ PAH 4	∑ PAH 8
**Fully fermented tea (*n* = 17)**											
Mean	12.67	<LOQ	4.42	<LOQ	<LOQ	ND	ND	ND	12.67	17.10	17.10
Minimum	<LOQ	ND	ND	ND	ND	ND	ND	ND	–	–	–
Maximum	57.38	<LOQ	50.56	<LOQ	<LOQ	ND	ND	ND	57.38	107.94	107.94
Detected Frequency (%)	100	58.8	11.8	11.8	17.6	0	0	0			

**Semi fermented tea (*n* = 6)**											
Mean	8.35	<LOQ	ND	ND	ND	ND	ND	ND	8.35	8.35	8.35
Minimum	<LOQ	ND	ND	ND	ND	ND	ND	ND	–	–	–
Maximum	50.12	<LOQ	ND	ND	ND	ND	ND	ND	50.12	50.12	50.12
Detected Frequency (%)	100	50	0	0	0	0	0	0			

**Non fermented tea (*n* = 6)**											
Mean	22.59	<LOQ	9.02	ND	<LOQ	ND	ND	ND	22.59	31.61	31.61
Minimum	<LOQ	ND	ND	ND	ND	ND	ND	ND	–	–	–
Maximum	45.79	<LOQ	54.12	ND	<LOQ	ND	ND	ND	45.79	99.91	99.91
Detected Frequency (%)	100	50	16.7	0	16.7	0	0	0			

**Post fermented tea (*n* = 6)**											
Mean	58.06	30.92	25.89	<LOQ	18.68	ND	10.73	ND	76.74	133.54	144.27
Minimum	<LOQ	<LOQ	ND	<LOQ	<LOQ	ND	ND	ND	–	–	–
Maximum	155.16	185.50	49.33	<LOQ	75.75	ND	64.35	ND	230.91	465.74	530.09
Detected Frequency (%)	100	100	66.67	66.67	66.67	0	16.67	0			

**Tisanes (*n* = 8)**											
Mean	<LOQ	<LOQ	4.28	<LOQ	<LOD	ND	ND	ND	<LOQ	4.28	4.28
Minimum	<LOQ	ND	ND	ND	ND	ND	ND	ND	–	–	–
Maximum	<LOQ	<LOQ	34.25	<LOQ	<LOD	ND	ND	ND	<LOQ	34.25	34.25
Detected Frequency (%)	100	12.5	12.5	12.5	0	0	0	0			

<LOD: below limit of detection; <LOQ: below limit of quantitation; ND: not detected.

ΣPAH2 = BaP + CHR.

ΣPAH4 = ΣPAH2 + BaA + BbFA.

ΣPAH8 = ΣPAH4 + BkFA + DBahA + BghiP + IP.

This trend is consistent with previous findings showing that dark tea (post-fermented tea) had the highest levels of PAHs contamination among tea categories (Ma et al., 2023). In addition, post-fermented tea is often produced from older mature leaves large-leaf cultivars, which have a relatively larger leaf surface area and may therefore retain more PAHs through atmospheric deposition (Giráldez et al., 2025; Zheng et al., 2026). Furthermore, high processing temperatures and prolonged baking times may also contributed to elevated PAH levels in post-fermented tea. Chiang et al. (2021) reported that PAHs concentrations in tea increased by more than 50% when roasting duration was increased from 4 h to 12 h. Similarly, Ziegenhals et al. (2008) and Schulz et al. (2014) reported the lowest PAHs contamination in tisanes, higher PAHs levels in non-fermented green tea than in fully fermented black tea. Rivera-Vera et al. (2019) also found higher total PAHs concentrations in green tea than in black tea. The variability in different types of tea could be due to different manufacturing processes such as withering, rolling, fermentation, and drying (Chiang et al., 2021; Guo et al., 2022; Rivera-Vera et al., 2019). Grover et al. (2013) reported that total PAHs concentration decreased during rolling and fermentation stages due to the volatilization and degradation of PAHs. However, total PAHs concentrations increased 10 times during drying stage when coal was used as the fuel source. On the other hand, Lin and Zhu (2004) also reported a 73-fold increase in total PAHs concentration in black tea compared to fresh tea leaves when pine firewood was used as the fuel source during the drying process. Rivera-Vera et al. (2019) also suggested that the variation in PAHs concentration among commercial brands within the same tea category may be associated with differences in plant physiological characteristics, including leaf surface morphology, cuticle thickness, and overall plant composition. In addition, geographic origin may also influence PAH contamination, as plantation practices, soil quality, water quality, and atmospheric deposition can differ across regions (Hu et al., 2025; Noori & Taşdemir, 2025). For instance, Ma et al. (2023) reported relatively higher PAH levels in tea from Hunan than in teas from other China provinces. This finding is consistent with Han et al. (2019), who reported higher PAHs concentrations in both urban and rural soils from Hunan compared with several other regions of China. Intensive coal use, biomass combustion, and vehicular emissions were suggested as the major contributors to PAH contamination in soils, with particulate-bound PAHs deposited onto the soil surfaces through atmospheric deposition (Han et al., 2019). The contribution of atmospheric deposition is further supported by Jia et al. (2019), that reported that 90.6% of PAHs in vegetables were absorbed through leaf stomata and surface, and only 9.4% were taken up from soil through roots. On the contrary, the relatively low PAHs levels observed in tisanes could be attributed to differences in manufacturing processes with traditional teas. Tisanes are generally produced from cultivated or wild-harvested plants that are gently dried, sorted and processed with minimal heat exposure, which may reduce PAHs formation or accumulation during production (Ciemniak et al., 2019). It is noteworthy that among the PAHs analyzed, the 4-ring compounds (CHR and BaA) were the most frequently detected. CHR was detected in all tea categories, with a detection rate of 100%, while BaA was detected in more than 50% of samples in each category, except tisanes, where its detection rate was markedly lower (12.5%). In contrast, among the forty-three samples analyzed, BghiP (6-ring PAH) was detected in only one sample (detection rate of 2.32%). The other 6-ring PAH (IP), along with the 5-ring PAH (DBahA) were not detected in any tea samples. This finding is consistent with those reported by Guo et al. (2022). The non-detection of these compounds maybe attributed to their relatively higher LOQs (70.04 ng L^−1^ for DBahA and 98.47 ng L^−1^ for IP) compared with other target PAHs (11.73–63.48 ng L^−1^). In addition, DBahA and IP are high-molecular weight PAHs with low volatility and strong affinity for particulate matter, including soot, dust, and other airborne particles. Their strong airborne particle-binding tendency may limit desorption from particulate surfaces, thereby reducing their environmental mobility and accumulation on tea leaves (Yang et al., 2017). Notably, BaP (5-ring PAH) recognized for its high carcinogenic potential, showed the highest detection rate in post-fermented tea and was not detected in tisanes and semi-fermented tea. Similarly, the other 5-ring PAHs (BbFa and BkFA), were frequently detected in post-fermented tea (66.67%), but were absent in semi-fermented tea. The detection rates for these compounds in fully fermented tea, non-fermented tea and tisanes were all below 20%.

### 3.6 Dietary risk assessment of PAHs

To estimate the average and maximum daily intake of BaP, PAH2, PAH4 and PAH8 through tea consumption, Malaysia's widely consumed pulled tea was used as a reference model. Based on data from the National Health and Morbidity Survey (NHMS) conducted in 2014, the mean daily tea consumption per individual in Malaysia is approximately 326.26 mL (Institute for Public Health (IPH), 2014). According to the preparation guidelines provided by the Ministry of Health Malaysia, a typical serving of pulled tea contains 5 g of tea leaves brewed in 200 mL of water (Nutritionistkkm, 2024). Using this ratio, the estimated daily intake of tea leaves by an average Malaysian adult is approximately 8.2 g (Tan et al., 2024). Using this consumption estimate, deterministic assessments of the average and maximum daily intake values were calculated for each tea category, as presented in Table 3. It should be noted that the present exposure assessment was based on PAH concentrations measured in plain tea infusions, whereas Malaysian pulled tea is commonly consumed with milk. Milk addition may influence the bioaccessibility of PAHs because these hydrophobic compounds can partition into lipid-rich phases during digestion, potentially affecting their release and absorption. Hack and Selenka (1996) reported that lyophilized milk increased PAHs mobilization, while Zhang et al. (2015) found that non-fat milk did not significantly affect PAHs bioaccessibility. Therefore, the actual bioaccessible fraction of PAHs in milk-containing tea beverages may differ from estimates based solely on plain tea infusions. Currently, no official tolerable daily intake (TDI) has been established for PAHs because of their genotoxic and carcinogenic properties. Nevertheless, the European Food Safety Authority (EFSA) recommends the use of the margin of exposure (MOE) approach for risk characterization (European Food Safety Authority, 2008). MOE values derived from BaP, PAH2, PAH4, and PAH8 are considered useful and effective indicators for assessing potential genotoxic and carcinogenic risk (European Food Safety Authority, 2005). According to EFSA guidelines, MOE values approaching or below 10,000 may indicate a potential health concern for consumers and may warrant the need for risk management action (European Food Safety Authority, 2008). In this study, MOE values calculated using both average and highest EDI exceeded 7,000,000 for BaP and PAH8, and 6,000,000 for PAH2 and PAH4. These results suggest that dietary exposure to PAHs through tea consumption among Malaysian consumers is low and is unlikely to pose a significant health risk under the assessed consumption scenario. This finding is consistent with studies from other countries (China, Taiwan, Korea, Vietnam, and Poland), as summarized in Table S4 (Supplementary Information), where PAHs exposure through tea consumption was also considered low health risk, with MOE values exceeding 10,000.

**Table 3:** Dietary exposure and risk assessment of BaP, PAH2, PAH4, and PAH8 in different categories of tea samples.

Tea Category	PAHs	Detected Concentration (ng kg^−1^)		Estimated daily intake (EDI) (mg/kg BW/day)		BMDL_10_ (mg/kg BW/day)	Margin of Exposure (MOE)	
		Mean	Maximum	Mean	Maximum		Mean	Maximum
Fully fermented tea	BaP	0	0	0	0	0.07	0	0
	PAH2	12.67	57.38	1.48 × 10^−9^	6.72 × 10^−9^	0.17	1.15 × 10^8^	2.53 × 10^7^
	PAH4	17.10	107.94	2.00 × 10^−9^	1.26 × 10^−8^	0.34	1.70 × 10^8^	2.69 × 10^7^
	PAH8	17.10	107.94	2.00 × 10^−9^	1.26 × 10^−8^	0.49	2.45 × 10^8^	3.88 × 10^7^

Semi fermented tea	BaP	0	0	0	0	0.07	0	0
	PAH2	8.35	50.12	9.78 × 10^−10^	5.87 × 10^−9^	0.17	1.74 × 10^8^	2.90 × 10^7^
	PAH4	8.35	50.12	9.78 × 10^−10^	5.87 × 10^−9^	0.34	3.48 × 10^8^	5.79 × 10^7^
	PAH8	8.35	50.12	9.78 × 10^−10^	5.87 × 10^−9^	0.49	5.01 × 10^8^	8.35 × 10^7^

Non fermented tea	BaP	0	0	0	0	0.07	0	0
	PAH2	22.59	45.79	2.65 × 10^−9^	5.36 × 10^−9^	0.17	6.42 × 10^7^	3.17 × 10^7^
	PAH4	31.61	99.91	3.70 × 10^−9^	1.17 × 10^−8^	0.34	9.18 × 10^7^	2.91 × 10^7^
	PAH8	31.61	99.91	3.70 × 10^−9^	1.17 × 10^−8^	0.49	1.32 × 10^8^	4.19 × 10^7^

Post fermented tea	BaP	18.68	75.75	2.19 × 10^−9^	8.87 × 10^−9^	0.07	3.20 × 10^7^	7.89 × 10^6^
	PAH2	76.74	230.91	8.99 × 10^−9^	2.70 × 10^−8^	0.17	1.89 × 10^7^	6.28 × 10^6^
	PAH4	133.54	465.74	1.56 × 10^−8^	5.46 × 10^−8^	0.34	2.17 × 10^7^	6.23 × 10^6^
	PAH8	144.27	530.09	1.69 × 10^−8^	6.21 × 10^−8^	0.49	2.90 × 10^7^	7.89 × 10^6^

Tisanes	BaP	0	0	0	0	0.07	0	0
	PAH2	0	0	0	0	0.17	0	0
	PAH4	4.28	34.25	5.01 × 10^−10^	4.01 × 10^−10^	0.34	6.78 × 10^8^	8.47 × 10^7^
	PAH8	4.28	34.25	5.01 × 10^−10^	4.01 × 10^−10^	0.49	9.77 × 10^8^	1.22 × 10^8^

PAH2: CHR and BaP; PAH4: PAH2, BbFA, and BaA; PAH8: PAH4, BkFA, DBahA, BghiP and IP.

## 4 Conclusion

In this study, an improved DLLME-UPLC-FLD method was developed and validated for the simultaneous extraction, pre-concentration, separation, and quantification of eight EFSA-recommended PAHs in tea. The method demonstrated satisfactory analytical performance and was successfully applied to determine PAHs in tea products marketed in Malaysia. The results showed that PAHs contamination levels varied across tea categories, with post-fermented teas exhibiting the highest mean total PAHs content. However, the margin of exposure assessment indicated that PAHs exposure through tea consumption is unlikely to pose a significant health risk to the Malaysian population. Nevertheless, this study was limited to tea products available in Malaysia, and focused only on the eight EFSA-recommended PAHs. Future studies should include a larger cohort of tea samples from different geographical regions and investigate a wider range of PAHs to provide a more comprehensive assessment of contamination patterns and consumer exposure. In addition, further investigation into the influence of milk addition on PAH bioaccessibility in milk-containing tea beverages is warranted. Overall, the proposed method offers a rapid, sensitive, and environmentally friendly analytical approach for routine PAHs analysis in tea samples and supports efforts to ensure tea safety and consumer protection.

## CRediT authorship contribution statement

**Kim Liu Tan:** Writing – original draft, Validation, Project administration, Methodology, Investigation, Formal analysis, Data curation. **Jalal T. Althakafy:** Writing – review & editing, Funding acquisition. **Yin-Hui Leong:** Writing – review & editing. **Yong Shen Chua:** Writing – review & editing, Supervision. **Faiz Bukhari Mohd Suah:** Writing – review & editing, Supervision. **Yong Foo Wong:** Writing – review & editing, Supervision, Resources, Investigation, Conceptualization.

## Funding statement

This research work was funded by Umm Al-Qura University, Saudi Arabia under grant number: 26UQU4281758GSSR02.

## Declaration of competing interest

The authors declare that they have no known competing financial interests or personal relationships that could have appeared to influence the work reported in this paper.

## Data Availability

Data will be made available on request.
