# Supplementary material for: Dispersive liquid-liquid microextraction coupled with ultra performance liquid chromatography-fluorescence detection for the determination of polycyclic aromatic hydrocarbons in tea: Method development, validation, greenness assessment, and dietary exposure risk assessment
Source: Food Chem X. 2026 Aug 29;39:104387. doi: 10.1016/j.fochx.2026.104387 (PMC13563586; doi:10.1016/j.fochx.2026.104387)
Supplement: Supplementary file 1 [file mmc1.pdf]

Dispersive liquid-liquid microextraction coupled with ultra performance liquid chromatography-fluorescence detection for the determination of polycyclic aromatic hydrocarbons in tea: Method development, validation, greenness assessment, and dietary exposure risk assessment

Kim Liu Tan[a], Jalal T. Althakafy[b], Yin-Hui Leong[c], Yong Shen Chua[d], Faiz Bukhari Mohd Suah[d], Yong Foo Wong[a,*]

[a] Center for Research on Multidimensional Separation Science, School of Chemical Sciences, Universiti Sains Malaysia, 11800 Penang, Malaysia

[b] Department of Chemistry, Faculty of Science, Umm Al-Qura University, Makkah 21955, Saudi Arabia

[c] National Poison Centre, Universiti Sains Malaysia, 11800 Penang, Malaysia

[d] School of Chemical Sciences, Universiti Sains Malaysia, 11800 Penang, Malaysia

Submission to

Food Chemistry: X

* Corresponding author

Tel; +604 653 4031

Fax: +604 657 4854

E-mail: wongyongfoo@usm.my (Y.F. Wong)

Table S1 PAH contents (mean ± SD) in tea determined using the validated DLLME-UPLC-FLD method.

Tea Samples	Country of Origin	Average concentration ± SD (ng kg^{-1})								∑ PAH 2 (ng kg^{-1})	∑ PAH 4 (ng kg^{-1})	∑ PAH 8 (ng kg^{-1})
		CHR	BaA	BbFA	BkFA	BaP	DBahA	BghiP	IP			
**Fully fermented**												
Black tea 1 (BT1)	Malaysia	< LOQ	< LOD	ND	ND	ND	ND	ND	ND	-	-	-
Black tea 2 (BT2)	Malaysia	38.14 ± 6.34	< LOQ	ND	ND	< LOQ	ND	ND	ND	38.14	38.14	38.14
Black tea 3 (BT3)	Indonesia	< LOQ	< LOD	ND	ND	< LOD	ND	ND	ND	-	-	-
Black tea 4 (BT4)	Arab	< LOQ	< LOQ	ND	ND	ND	ND	ND	ND	-	-	-
Black tea 5 (BT5)	UK	< LOQ	< LOD	ND	ND	ND	ND	ND	ND	-	-	-
Black tea 6 (BT6)	Malaysia	< LOQ	< LOQ	ND	ND	ND	ND	ND	ND	-	-	-
Black tea 7 (BT7)	China	< LOQ	< LOQ	ND	ND	ND	ND	ND	ND	-	-	-
Black tea 8 (BT8)	Malaysia	< LOQ	< LOD	ND	ND	ND	ND	ND	ND	-	-	-
Black tea 9 (BT9)	Sri Lanka	57.38 ± 9.81	< LOQ	ND	ND	ND	ND	ND	ND	57.38	57.38	57.38
Black tea 10 (BT10)	Singapore	< LOQ	< LOQ	ND	ND	ND	ND	ND	ND	-	-	-
Black tea 11 (BT11)	Sri Lanka	< LOQ	< LOD	ND	ND	ND	ND	ND	ND	-	-	-
Black tea 12 (BT12)	Arab	39.79 ± 4.45	< LOQ	ND	ND	ND	ND	ND	ND	39.79	39.79	39.79
Black tea 13 (BT13)	Sri Lanka	< LOQ	< LOQ	ND	ND	ND	ND	ND	ND	-	-	-
Red tea 1 (RT1)	China	< LOQ	< LOD	ND	ND	ND	ND	ND	ND	-	-	-
Red tea 2 (RT2)	Taiwan	42.35 ± 7.57	< LOQ	50.56 ± 5.50	< LOQ	< LOQ	ND	ND	ND	42.35	92.91	92.91
Flavored black tea 1 (FBT1)	Malaysia	37.81 ± 5.36	< LOQ	24.63 ± 7.04	< LOQ	< LOQ	ND	ND	ND	37.81	62.44	62.44
Flavored black tea 2 (FBT2)	Arab	< LOQ	< LOD	ND	ND	ND	ND	ND	ND	-	-	-
**Semi fermented**												
Tie Guan Yin 1 (TGY1)	China	< LOQ	< LOQ	ND	ND	ND	ND	ND	ND	-	-	-
Tie Guan Yin 2 (TGY2)	China	< LOQ	< LOQ	ND	ND	ND	ND	ND	ND	-	-	-
Tie Guan Yin 3 (TGY3)	China	< LOQ	< LOD	ND	ND	ND	ND	ND	ND	-	-	-
Oolong 1 (O1)	Japan	< LOQ	< LOD	ND	ND	ND	ND	ND	ND	-	-	-
Oolong 2 (O2)	Singapore	50.12 ± 9.10	< LOQ	ND	ND	ND	ND	ND	ND	50.12	50.12	50.12
Da Hong Pao (DHP)	China	< LOQ	< LOD	ND	ND	ND	ND	ND	ND			
**Non fermented**												
Green tea 1 (GT1)	Japan	< LOQ	< LOD	ND	ND	ND	ND	ND	ND	-	-	-
Green tea 2 (GT2)	Malaysia	< LOQ	< LOD	ND	ND	ND	ND	ND	ND	-	-	-
Green tea 3 (GT3)	Poland	45.80 ± 7.22	< LOQ	ND	ND	ND	ND	ND	ND	45.80	45.80	45.80
Green tea 4 (GT4)	China	44.19 ± 7.22	< LOQ	ND	ND	< LOQ	ND	ND	ND	44.19	44.19	44.19
Flavored green tea (FGT1)	Arab	< LOQ	< LOD	54.12 ± 12.9	ND	ND	ND	ND	ND	-	54.12	54.12

Flavored green tea (FGT2)	Sri Lanka	45.56 ± 6.7	< LOQ	ND	ND	ND	ND	ND	ND	45.56	45.56	45.56
**Post fermented**												
Pu Erh Raw (PER)	China	< LOQ	< LOQ	ND	ND	ND	ND	ND	ND	-	-	-
Pu Erh 1 (PE1)	China	42.12 ± 6.04	< LOQ	33.76 ± 6.95	< LOQ	< LOQ	ND	ND	ND	42.12	75.88	75.88
Pu Erh 2 (PE2)	Sri Lanka	38.30 ± 7.65	< LOQ	ND	ND	ND	ND	ND	ND	38.30	38.30	38.30
Dark tea (DT)	China	40.80 ± 6.98	< LOQ	34.47 ± 10.44	< LOQ	< LOQ	ND	ND	ND	40.80	75.27	75.27
Liu Bao Tea (LBT)	China	71.98 ± 7.98	< LOQ	37.79 ± 9.82	< LOQ	36.32 ± 6.27	ND	ND	ND	108.30	146.09	146.09
Oriental Delight tea (FPE)	Taiwan	155.15 ± 12.48	185.50 ± 3.90	49.33 ± 11.84	< LOQ	75.74 ± 9.37	ND	64.35 ± 40.36	ND	230.89	465.72	530.07
**Tisanes**												
Lemon Myrtle (T1)	Malaysia	< LOQ	< LOD	ND	ND	ND	ND	ND	ND	-	-	-
Camomile 1 (T2)	Malaysia	< LOQ	< LOD	ND	ND	ND	ND	ND	ND	-	-	-
Camomile 2 (T3)	UK	< LOQ	< LOQ	34.25 ± 6.51	< LOQ	< LOD	ND	ND	ND	-	34.25	34.25
Detox tea (T4)	Arab	< LOQ	< LOD	ND	ND	ND	ND	ND	ND	-	-	-
Peppermint 1 (T5)	UK	< LOQ	< LOD	ND	ND	ND	ND	ND	ND	-	-	-
Peppermint 2 (T6)	UK	< LOQ	< LOD	ND	ND	ND	ND	ND	ND	-	-	-
Pecah Beling (T7)	Malaysia	< LOQ	< LOD	ND	ND	ND	ND	ND	ND	-	-	-
Orthosiphon aristatus (T8)	Malaysia	< LOQ	< LOD	ND	ND	ND	ND	ND	ND	-	-	-

<LOD: below limit of detection; <LOQ: below limit of quantitation; ND: not detected.

ΣPAH2 = BaP + CHR

ΣPAH4 = ΣPAH2 + BaA + BbFA

ΣPAH8 = ΣPAH4 + BkFA + DBahA + BghiP + IP

Table S2 Adopted UPLC-FLD conditions.

Variable	Optimum value
Flow rate (mL/ min)	0 – 1 min: 0.45<br>1 – 10 min: 0.75 (backpressure ≈ 937.5 bar)<br>10 – 19 min: 0.75<br>19 – 20 min: 0.45<br>20 – 25 min: 0.45
Temperature (°C)	35
Initial mobile phase composition (%)	ACN:UPW (60:40)
Gradient programming<br>(ACN composition, %)	0 – 1 min (60 %)<br>1 – 10 min (63.6 %)<br>10 – 13 min (64.8 %)<br>13 – 14 min (100 %)<br>14 – 19 min (100 %)<br>19 – 20 min (60 %)<br>20 – 25 min (60 %)
Injection volume (μL)	5
FLD configuration	Excitation (nm): 270<br>Emission (nm): 385, 405, 420, 510

Table S3 Comparison of LC-based analytical methods for the determination of PAHs in tea samples.

Analyte	Sample Preparation	Extraction time (min)	LOD	LOQ	Recovery	Enrichment Factor (EF)	Analysis method	References
BaA, CHR, BbFA, BkFA, BaP, DBahA	HDES-DLLME	11	5.5 – 38.8 ng L^{-1}	18.6 – 129.1 ng L^{-1}	62.5% – 91.8%	33 - 49	UPLC-FLD	Mesías-Salazar et al. (2023)
BaA, CHR, BbFA, BkFA, BaP, DBahA	IL-DLLME	< 25	2.02-28.6 ng L^{-1}	6.73 – 95.2 ng L^{-1}	57.0% – 94.0 %	61 - 94	HPLC-FLD	Rivera-Vera et al. (2019
Nap, Flu, Phe, Ant, Flt, Pyr, BaA, CHR, BbFA, BkFA, BaP, DBahA, and BghiP	SA-DLLME	4	0.02 – 0.04 μg L^{-1}	0.05 – 0.11 μg L^{-1}	85% – 105%	37 - 46	HPLC-FLD	Timofeeva et al. (2021)
BaA, CHR, BaP, BbFA, BkFA	MIL-DLLME	18	0.005 – 0.02 μg L^{-1}	0.02 – 0.05 μg L^{-1}	89.8% - 120%	8.84 - 16.6	HPLC-FLD	Trujillo-Rodríguez et al. (2016)
Nap, BP, Flu, Phe, Ant	AA-LLME-SFDES	10	0.16 – 0.75 μg L^{-1}	0.5 – 2.5 μg L^{-1}	82.0% – 116.6%	17 - 18	HPLC-UV	Zhang et al. (2021)

Table S3 Continued

Analyte	Sample Preparation	Extraction time (min)	LOD	LOQ	Recovery	Enrichment Factor (EF)	Analysis method	References
BaA, CHR, BaP, BbFA	Online SPE	32 (include detection)	0.05 – 0.15 μg L^{-1}	0.15 – 0.50 μg L^{-1}	83.0% – 107.0%	-	HPLC-UV-Vis	Siritham et al. (2017)
Nap, Ace, Flu, Phe, Ant, Fla, Pyr, BaA, CHR, BbFA, BkFA, BaP, DBahA, BghiP, IP and BeP	MASE	70	0.6 – 28.2 ng L^{-1}	1.0 – 39.2 ng L^{-1}	76.0 % – 117%	42.8	HPLC-FLD	Mañana-López et al. (2021)
Flu, Ant, Pyr, BbFA, BaP	SMME	15	0.02 – 0.08 μg L^{-1}	0.07 – 0.23 μg L^{-1}	85.5% – 102.1%	-	HPLC-FLD	Zhou et al. (2021)
BaA, BbFA, BaP	VA-SPE	1.5	1.65 – 2.06 ng L^{-1}	5.5 – 6.9 ng L^{-1}	70.1% – 119.3%	-	HPLC-DAD	Myint Zaw et al. (2022
BaA, CHR, BbFA, BkFA, BaP, DBahA, BghiP, and IP	DLLME	< 10	3.87 – 32.5 ng L^{-1}	11.73 – 98.47 ng L^{-1}	85.13% – 101.71%	35 - 48	UPLC-FLD	This study

AA-LLME-SFDES, Air-assisted liquid-liquid microextraction solidification of floating deep eutectic solvents; Ace, acenaphthene; Ant, anthracene; BaA, benzo(a)anthracene; BaP, benzo(a)pyrene; BbFA, benzo(b)fluoranthene; BeP, benzo(e)pyrene; BghiP, benzo(g,h,i)perylene; BkFA, benzo(k)fluoranthene; BP, Biphenyl; CHR, chrysene; DAD, diode array detector; DBahA, dibenz(a,h)anthracene; DLLME, dispersive liquid-liquid microextraction; FLD, fluorescence detection; Flu, fluorene; Fla, fluoranthene; HDES, hydrophobic deep eutectic solvent; HPLC, high performance liquid chromatography; IL, ionic liquid; IP, indeno(1,2,3-cd)pyrene; MASE, membrane assisted solvent extraction; MIL, magnetic ionic liquid; Nap, naphthalene; Phe, phenanthrene; Pyr, pyrene; SA, surfactant-assisted; SMME, syringe membrane-based microextraction; SPE, solid phase extraction; UHPLC, ultra-high performance liquid chromatography; UV, ultraviolet; VA, vortex-assisted; Vis, visible.

Table S4 Comparison of PAH4 and PAH8 margin of exposure (MOE) values in tea from this study and other reported studies.

**Country**	**PAH**	**MOE range**	**Reference**
Malaysia	PAH4	6,230,000 – 84,700,000	This study
	PAH8	7,890,000 – 122,000,000	
China	PAH8	15,655 – 53,683	Liao et al. (2023)
Taiwan	PAH4	172,000 – 296,000	Harrison et al. (2024)
Korea	PAH4	4,430,000 – 4,630,000	Lee et al. (2018)
Vietnam	PAH4	1,270,642 – 2,391,604	Phan Thi et al. (2020)
Poland	PAH4	400,000	Roszko et al. (2018)
Poland	PAH4	830,000	Ciemniak et al. (2019)

PAH4: BAP, CHR, BbFA, BaA

PAH8: PAH4 + BkFA, DBahA, BghiP and IP

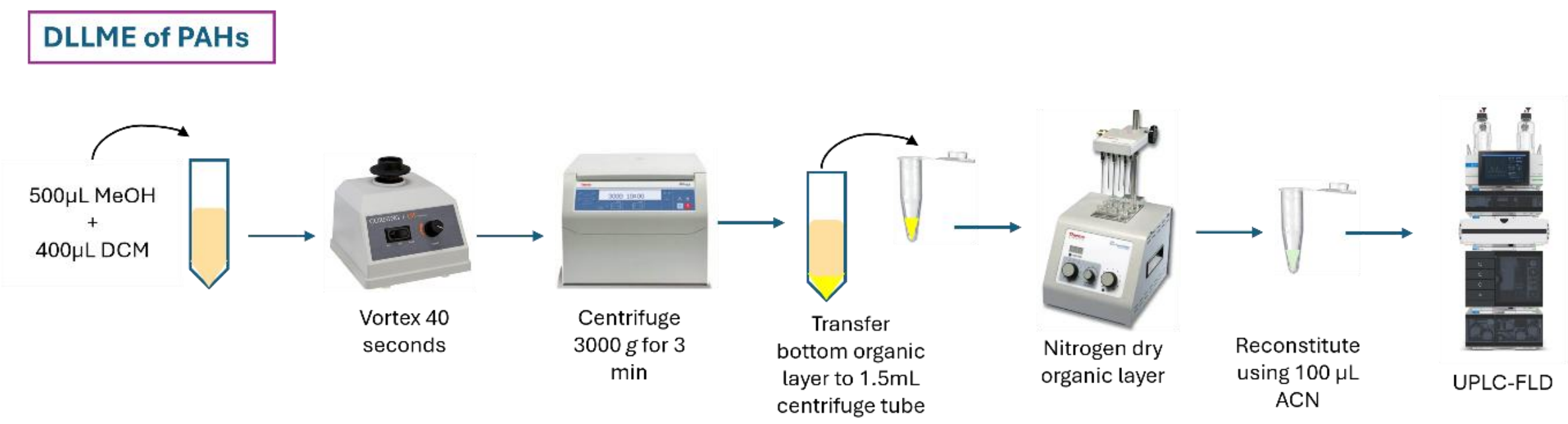

Figure S1 Schematic representation of the dispersive liquid–liquid microextraction (DLLME) procedure used for the extraction of PAHs from tea samples prior to UPLC–FLD analysis.

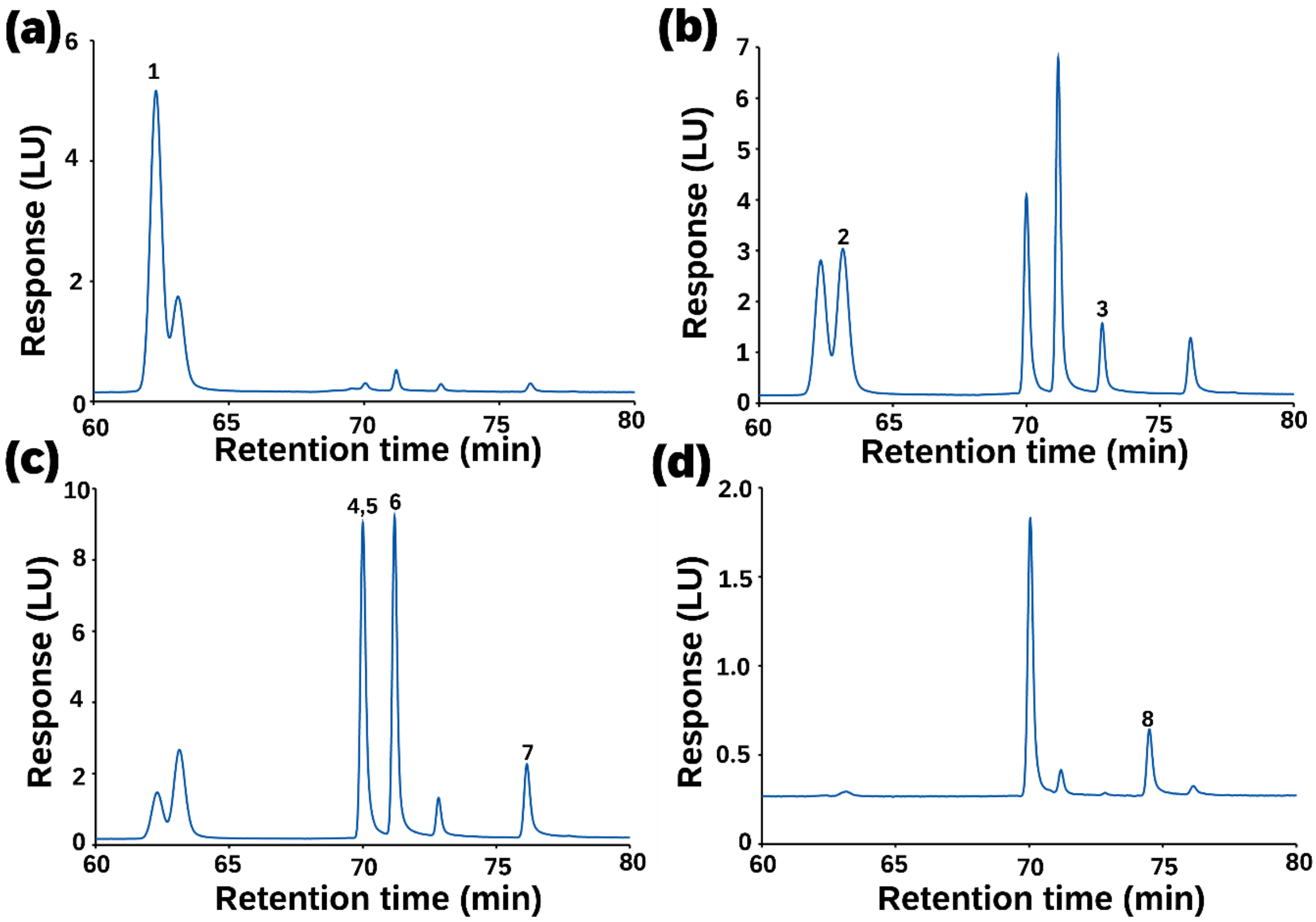

Figure S2 Chromatograms at (a) 385 nm, (b) 405 nm, (c) 420 nm, and (d) 510 nm for standards: 1, CHR; 2, BaA; 3, DBahA; 4, BbFA; 5, BkFA; 6, BaP; 7, BghiP; 8, IP. Analysis was performed using a PFP column (2.1 × 100 mm, 1.9 μm) with a flow rate of 0.2 mL/min and a column temperature of 25 °C. The elution program started with an isocratic hold at 30% ACN for 5 minutes, followed by a ramp to 50% ACN over 60 minutes, then to 62.5% ACN in 1 minute, to 67.5% ACN in 20 minutes, and finally a ramp to 100% ACN over 4 minutes.

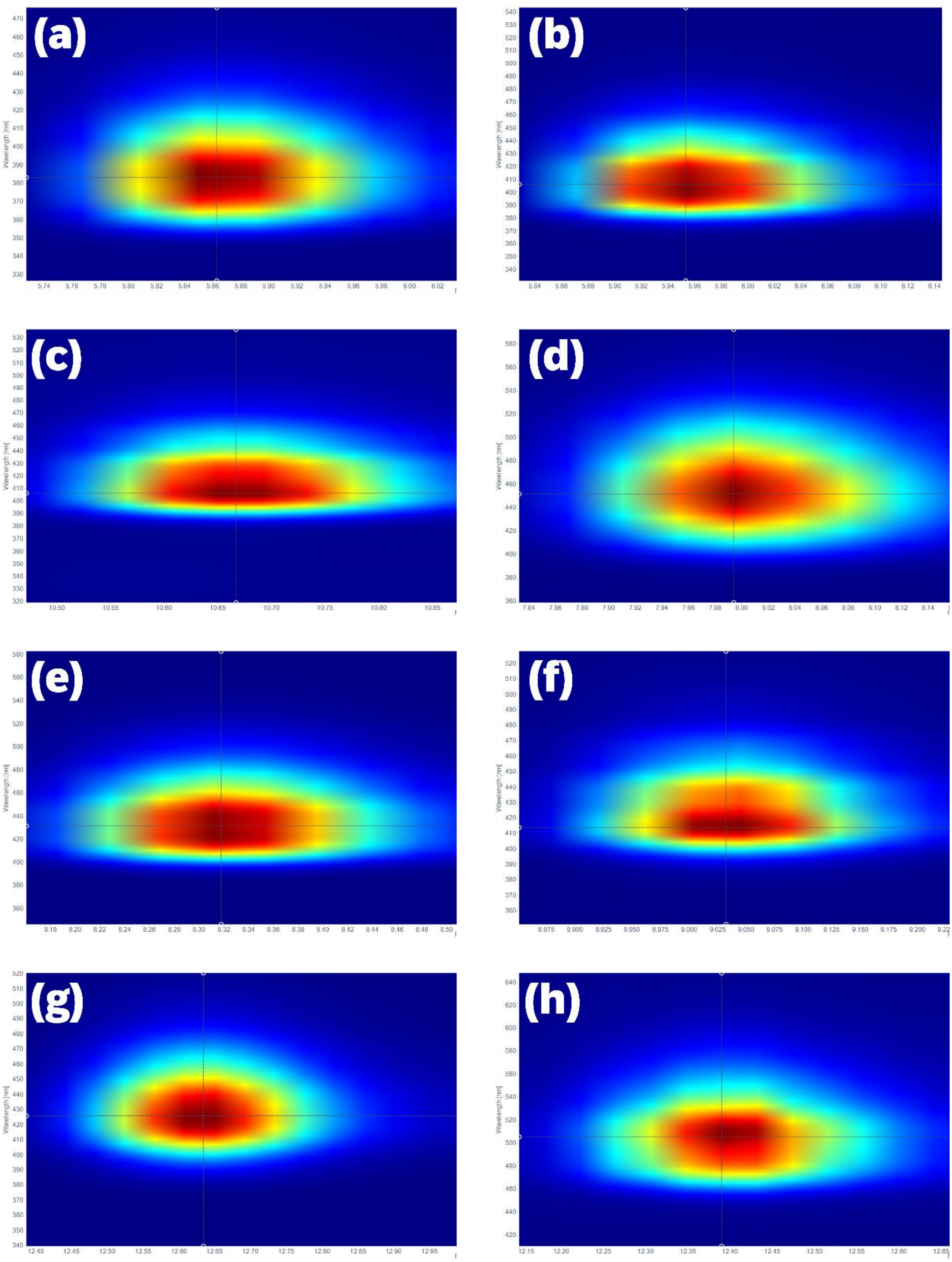

Figure S3 Fluorescence emission scan spectra of (a) CHR; (b) BaA; (c) DBahA; (d) BbFA; (e) BkFA; (f) BaP; (g) BghiP; (h) IP; at an excitation wavelength of 230 nm.

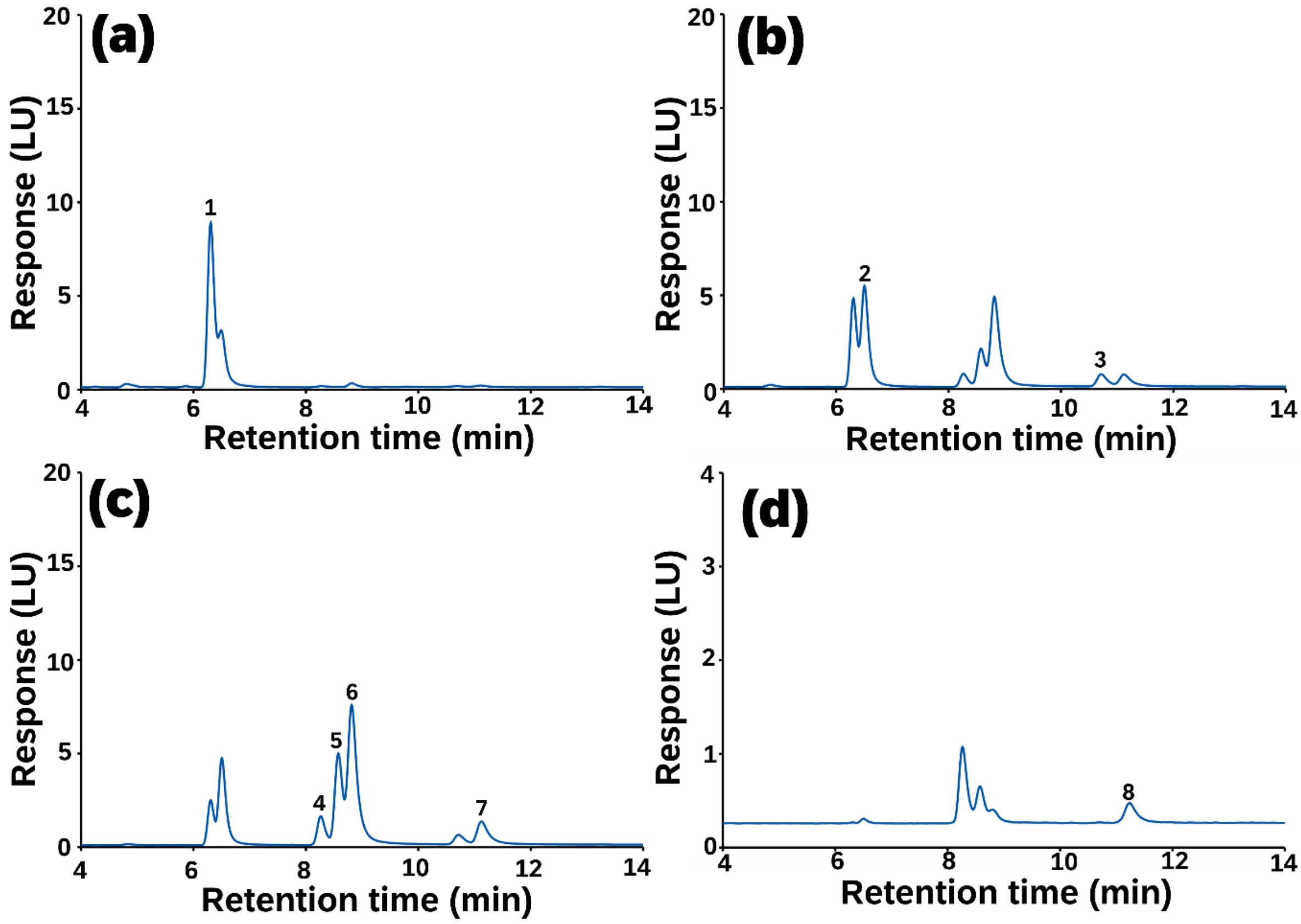

Figure S4 UPLC–FLD chromatograms of the PAH standard mixtures (0.02 mg L^{-1}) acquired at emission wavelengths of (a) 385nm; (b) 405nm; (c) 420nm; (d) 510nm. Peak assignments: 1, CHR; 2, BaA; 3, DBahA, 4, BbFA; 5, BkFA; 6, BaP; 7, BghiP; 8, IP.

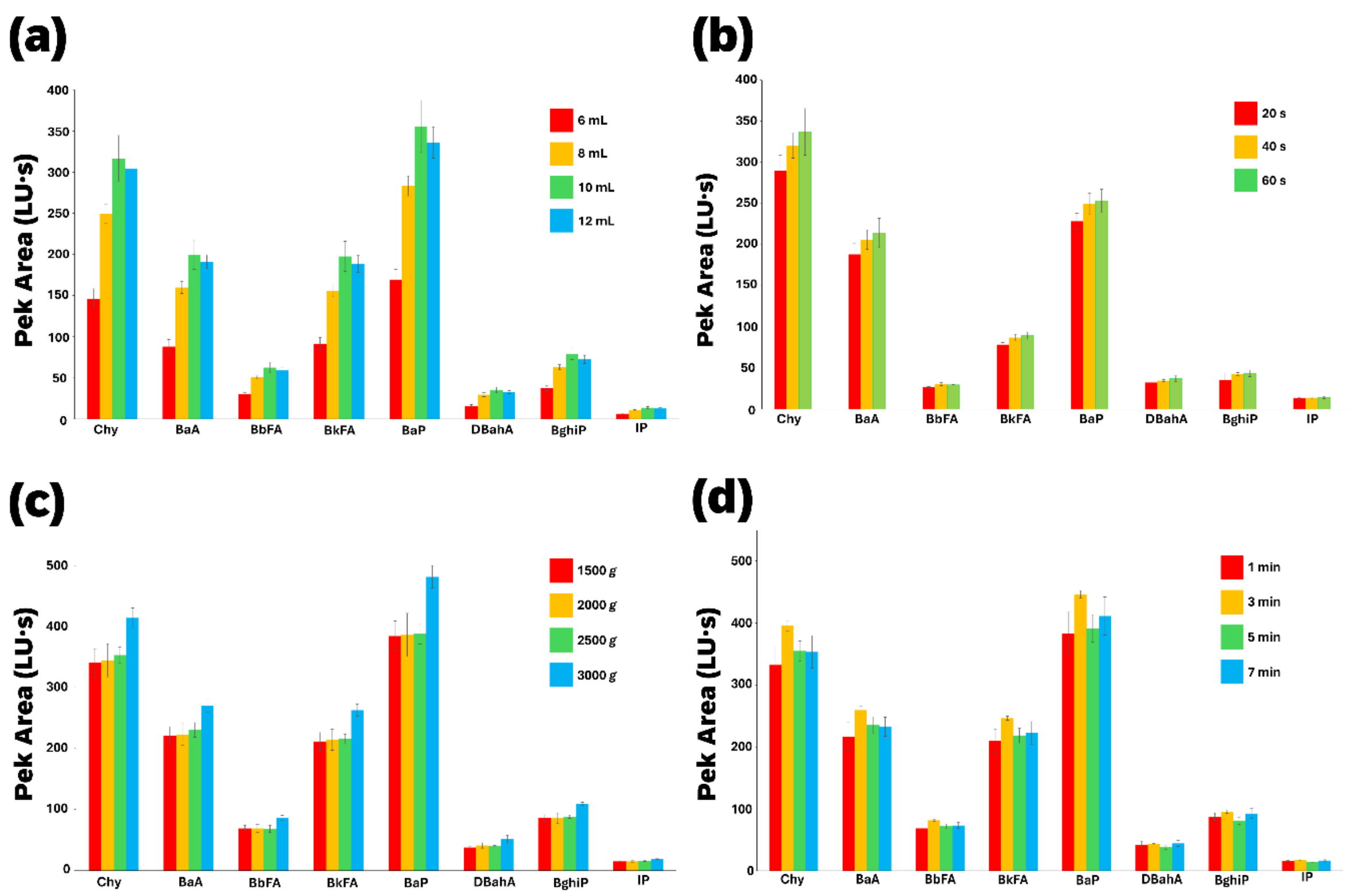

Figure S5 Effects of (a) sample volume, (b) vortex duration, (c) centrifugation speed, and (d) centrifugation duration on the extraction performance of PAHs.

References

Ciemniak, A., Kuźmicz, K., Rajkowska-Myśliwiec, M., & Cadena, M. F. (2019). Assessing the contamination levels of dried teas and their infusions by polycyclic aromatic hydrocarbons (PAHs). *Journal of Consumer Protection and Food Safety*, *14*(3), 263-274. https://doi.org/10.1007/s00003-019-01229-1

Harrison, D. M., Chang, W.-C., & Lin, H.-T. (2024). Dietary Exposure and Health Risk Assessment of Polycyclic Aromatic Hydrocarbons in Black Tea Consumed in Taiwan. *Toxics*, *12*(2), 134. https://doi.org/10.3390/toxics12020134

Lee, J.-G., Lim, T., Kim, S.-H., Kang, D.-H., & Yoon, H.-J. (2018). Determination and risk characterization of polycyclic aromatic hydrocarbons of tea by using the Margin of Exposure (MOE) approach. *Food Science and Biotechnology*, *27*(6), 1843-1856. https://doi.org/10.1007/s10068-018-0400-7

Liao, Z., Gao, Z., Cao, D., & Yang, Q. (2023). Occurrence and exposure evaluation of polycyclic aromatic hydrocarbons in tea samples consumed in Beijing-Tianjin-Hebei regions of China. *Journal of Food Composition and Analysis*, *123*, 105606. https://doi.org/10.1016/j.jfca.2023.105606

Mañana-López, A., Sánchez-Piñero, J., Moreda-Piñeiro, J., Turnes-Carou, I., Muniategui-Lorenzo, S., & López-Mahía, P. (2021). Polycyclic aromatic hydrocarbons analysis in tea infusions and tea beverages using membrane assisted solvent extraction. *Microchemical Journal*, *167*, 106278. https://doi.org/10.1016/j.microc.2021.106278

Mesías-Salazar, A., Rebolledo-Robles, K., Salazar-González, R., Bravo, M. A., Lucena, R., & Toledo-Neira, C. (2023). Hydrophobic deep eutectic solvent as extractant phase for determining six carcinogenic polycyclic aromatic hydrocarbons in tea and coffee infusion samples. *Microchemical Journal*, *193*, 109022. https://doi.org/10.1016/j.microc.2023.109022

Myint Zaw, M., Poorahong, S., Kanatharana, P., Thavarungkul, P., & Thammakhet-Buranachai, C. (2022). A simple gelatin aerogel tablet sorbent for the effective vortex assisted solid phase extraction of polycyclic aromatic hydrocarbons from tea samples. *Food Chemistry*, *383*, 132388. https://doi.org/10.1016/j.foodchem.2022.132388

Phan Thi, L.-A., Ngoc, N. T., Quynh, N. T., Thanh, N. V., Kim, T. T., Anh, D. H., & Viet, P. H. (2020). Polycyclic aromatic hydrocarbons (PAHs) in dry tea leaves and tea infusions in Vietnam: contamination levels and dietary risk assessment. *Environmental Geochemistry and Health*, *42*(9), 2853-2863. https://doi.org/10.1007/s10653-020-00524-3

Rivera-Vera, C., Lasarte-Aragonés, G., Bravo, M. A., Muñoz-Lira, D., Salazar, R., & Toledo-Neira, C. (2019). Ionic liquids-based dispersive liquid-liquid microextraction for determination of carcinogenic polycyclic aromatic hydrocarbons in tea beverages: Evaluation of infusion preparation on pollutants release. *Food Control*, *106*, 106685. https://doi.org/10.1016/j.foodcont.2019.06.011

Roszko, M., Kamińska, M., Szymczyk, K., & Jędrzejczak, R. (2018). Dietary risk evaluation for 28 polycyclic aromatic hydrocarbons (PAHs) in tea preparations made of teas available on the Polish retail market. *Journal of Environmental Science and Health, Part B*, *53*(1), 25-34. https://doi.org/10.1080/03601234.2017.1369323

Siritham, C., Thammakhet-Buranachai, C., Thavarungkul, P., & Kanatharana, P. (2017). A preconcentrator-separator two-in-one online system for polycyclic aromatic hydrocarbons analysis. *Talanta*, *167*, 573-582. https://doi.org/10.1016/j.talanta.2017.02.035

Timofeeva, I., Stepanova, K., & Bulatov, A. (2021). In-a-syringe surfactant-assisted dispersive liquid-liquid microextraction of polycyclic aromatic hydrocarbons in supramolecular solvent from tea infusion. *Talanta*, *224*, 121888. https://doi.org/10.1016/j.talanta.2020.121888

Trujillo-Rodríguez, M. J., Nacham, O., Clark, K. D., Pino, V., Anderson, J. L., Ayala, J. H., & Afonso, A. M. (2016). Magnetic ionic liquids as non-conventional extraction solvents for the determination of polycyclic aromatic hydrocarbons. *Analytica Chimica Acta*, *934*, 106-113. https://doi.org/10.1016/j.aca.2016.06.014

Zhang, K., Wang, Y., Li, S., & Zhu, G. (2021). Air-assisted liquid-liquid microextraction based on the solidification of floating deep eutectic solvents for the simultaneous determination of bisphenols and polycyclic aromatic hydrocarbons in tea infusions via HPLC. *Food Chemistry*, *348*, 129106. https://doi.org/10.1016/j.foodchem.2021.129106

Zhou, P., Zhang, W., & Wang, X. (2021). Development of a syringe membrane-based microextraction method based on metal–organic framework mixed-matrix membranes for preconcentration/extraction of polycyclic aromatic hydrocarbons in tea infusion. *Food Chemistry*, *361*, 130105. https://doi.org/10.1016/j.foodchem.2021.130105
